# Compartmentalisation of Hepatitis B virus X gene evolution in hepatocellular carcinoma microenvironment and the genotype-phenotype correlation of tumorigenicity in HBV-related patients with hepatocellular carcinoma

**DOI:** 10.1080/22221751.2022.2125344

**Published:** 2022-10-26

**Authors:** Ya Fu, Fengling Fang, Hongyan Guo, Xialin Xiao, Yuhai Hu, Yongbin Zeng, Tianbin Chen, Songhang Wu, Ni Lin, Jinlan Huang, Ling Jiang, Qishui Ou, Can Liu

**Affiliations:** a Department of Laboratory Medicine, The First Affiliated Hospital, Fujian Medical University, Fuzhou, People’s Republic of China; b Clinical Laboratory Diagnostics, The First Clinical College, Fujian Medical University, Fuzhou, People’s Republic of China; c Fujian Key Laboratory of Laboratory Medicine, The First Affiliated Hospital, Fujian Medical University, Fuzhou, People’s Republic of China; d Gene Diagnosis Research Center, Fujian Medical University, Fuzhou, People’s Republic of China; e Department of Hepatobiliary Surgery, The First Affiliated Hospital, Fujian Medical University, Fuzhou, People’s Republic of China

**Keywords:** Hepatitis B virus, hepatocellular carcinoma, quasispecies, mutation, tumorigenicity

## Abstract

Hepatitis B virus (HBV) exists as quasispecies (QS). However, the evolutionary characteristics of haplotypes of HBV X gene in the hepatocellular carcinoma (HCC) microenvironment remain unclear. Mutations across X gene are essential for the tumorigenicity of HBV X protein (HBx). However, the functional phenotypes of many mutant HBx remain unknown. This study aims to compare the characteristics of X gene evolution between tumour and non-tumour tissues in HCC patients and investigate the tumorigenic phenotype of HBx harbouring mutation T81P/S101P/L123S. This study included 24 HCC patients. Molecular cloning of X gene was performed to analyse characteristics of haplotypes in liver tissues. HCC cell lines stably expressing wild-type or mutant HBx and subcutaneous tumour xenograft mouse model were used to assess HBx-T81P/S101P/L123S tumorigenicity. The mean heterogeneity of HBV QS across X gene in tumour tissues was lower than that in non-tumour tissues. A location bias was observed in X gene clones with genotype C or D in tumour tissues compared to those with genotype B. Mutations in genotype-C or – D clones were mainly clustered in the dimerization region and aa110-aa140 within the transactivation region. A novel mutation combination at residues 81, 101 and 123 was identified in tumour tissues. Further, HBx-T81P/S101P/L123S promotes cell proliferation and increases genomic instability, which was mediated by MYC. This study elucidates the compartmentalized evolution patterns of HBV X gene between intra tumour and non-tumour tissues in HCC patients and provides a new mechanism underlying HBV-driven hepatocarcinogenesis, suggesting a potential viral marker for monitoring HCC.

## Introduction

Hepatitis B virus (HBV) infection is a major global health problem. Approximately 257 million people worldwide and approximately 70 million in China are chronically infected with HBV[1, 2]. Chronic HBV infection can lead to chronic hepatitis B (CHB), cirrhosis or hepatocellular carcinoma (HCC), which reports approximately one million deaths annually due to HBV-related complications[3]. The HBV genome contains four open reading frames (ORF), among which the ORF X encodes HBV X (HBx) protein. Although the HBx protein has been reported to be strongly associated with HCC development, its underlying carcinogenic mechanism remains unclear[4]. Moreover, various studies have demonstrated that mutations across the HBV X gene are involved in the progression of HBV-related liver diseases[5, 6], providing a new perspective on the development of HCC. Therefore, understanding the evolution of the HBV X gene in the HCC microenvironment and investigating the tumour-promoting phenotypes of mutant HBx proteins could contribute to elucidating the pathogenesis of HBV-related HCC.

Owing to a high replication rate and absent proofreading capacity during reverse transcription, HBV exists as quasispecies (QS) containing a spectrum of viral haplotypes with distinct fitness levels in certain environments[7, 8]. Under drug or immune pressure, HBV QS change dynamically. Furthermore, certain viral haplotypes that adapt to the pressure changes gradually become predominant due to the competitive replication[9]. The evolution of HBV QS has been studied in various serum samples[10–13]; however, the liver is the organ specifically infected by HBV, hence, HBV QS in the liver better reflects the truth than that in the serum. Recently, the characteristic of HBV QS in the liver tissues of patients with HCC has been investigated, wherein the QS heterogeneity across the HBV PreS region or core promoter region between tumour and adjacent non-tumour tissues was observed[14, 15]. Considering the close relationship of the HBV X gene with HCC[5], it is vital to elucidate the distinct QS characteristics across the X gene between tumour and non-tumour tissues in patients with HCC.

Based on the fact that the nucleotide sequence diverges from the entire genome by 8%, HBV has been classified into 10 genotypes (A – J), among which genotypes B and C are the most prevalent in Asia and Oceania[16]. Additionally, genotypes A, D, and I are also reported in Asia[16]. However, differences in the pathogenic impact among genotypes exist. Genotype C has been reported to cause the delayed hepatitis B e antigen (HBeAg) seroconversion, possessing a higher risk for cirrhosis and HCC than genotype B in East Asia[17]. Genotype D is also reported to cause more severe liver diseases than genotype A[17]. Additionally, genotype I is likely to be associated with the development of HCC[17]. Nonetheless, the underlying mechanism of the different genotypes in the development of HCC remains unknown.

It has been widely accepted that mutations across the HBV X gene are closely associated with the progression of HBV-related liver diseases. Furthermore, mutations across the HBV X gene, such as the amino acid (aa) alterations of V5M, S42P, V88F, H94Y, V/I127D/N, K130M and V131I, displayed a significant upward trend associated with liver disease progression to HCC[6, 18–20]. Patients with double mutations K130M/V131I, triple mutations I127T/K130M/V131I or K130M/V131I/F132Y were reported to have an increased prevalence of HBV-related liver diseases progression to HCC[6]. However, studies on the effect of HBx genotypic mutation on its phenotype are scarce. Currently, despite the number of significant mutations found in previous studies, the phenotype-confirmed HBx mutations include COOH-terminal truncated mutation[21], triple mutation L129S/K130M/V131I[21], double mutation A10R/S144R[22] and single mutation F30V[23]. However, various potential mutant HBx proteins remain to be discovered and their tumour-promoting roles are yet to be identified, which is crucial in improving our knowledge of the carcinogenic mechanism of HBx.

This study comprehensively analyses the characteristics of HBV QS across the X gene in the liver microenvironment of patients with HCC and compares the difference in QS heterogeneity between intra-patient tumour and adjacent non-tumour tissues. Furthermore, we investigate the distribution of different genotypes in the intra-patient liver microenvironment in patients with HCC co-infected with multiple-genotype HBV. In addition, we also investigate the mechanism underlying the tumour-promoting effect of HBx harbouring a novel triple mutation T81P/S101P/L123S.

## Materials and methods

### Patients

A total of 24 patients with HCC and detectable HBV DNA were enrolled in this study. Fresh HCC samples and adjacent non-tumour tissues were obtained from 24 patients who had undergone routine surgery from 2016 to 2020 at the First Affiliated Hospital of Fujian Medical University. Tissues were frozen and stored in liquid nitrogen until further use. Written informed consent was obtained from each patient involved in the study, and the study was approved by the Ethics Committee of the First Affiliated Hospital of Fujian Medical University.

### Liver biochemistry, HBV serology and HBV DNA tests

Serum alanine aminotransferase (ALT), aspartate aminotransferase (AST), hepatitis B surface antigen (HBsAg), hepatitis B surface antibody (HBsAb), HBeAg, hepatitis B e antibody (HBeAb), hepatitis B core antibody (HBcAb), HBV DNA and alpha-fetoprotein (AFP) were quantified as described in a previous study[24]. Briefly, ALT and AST were determined using an automated biochemical system (Siemens Healthcare Diagnostics, USA). Serum HBsAg, HBsAb, HBeAg, HBeAb and HBcAb were measured using the automatic immune fluorescence analyser Abbott Type i4000 (Abbott Laboratories, USA), following the manufacturer’s instructions. Serum HBV DNA was quantified using the TaqMan polymerase chain reaction (PCR) assay (Sansure Biotech, China) in a Roche LightCycler 480 Real-Time PCR System (Roche, Switzerland) with a low quantification limit of 500 IU/ml.

### Molecular cloning and sequencing

The extraction of genomic DNA and amplification of the HBV X gene were performed according to a previous report[22]. PCR products comprising the HBV X gene were purified using the TIANgel Mini Purification Kit (DP208, TIANGEN, China), cloned into the pGEM-T vector (A1360, Promega, USA) and transformed into the Trans1-T1 Phage Resistant Chemically Competent Cell (CD501, Transgen, China) growing on ampicillin plates. The clones per sample were sequenced by the Beijing Genomics Institute in Shenzhen, China.

### Sequence analysis

Phylogenetic trees were constructed using MEGA 7.0 for analysing the evolution of HBV X QS in the liver tissues. Shannon entropy (Sn) was used to quantify the complexity of HBV X QS. Sn was calculated at both the nucleotide and aa levels according to the following formula: Sn = –∑i(pi×lnpi)/lnN, where i refers to the type of haplotype, pi refers to the proportion of a certain haplotype among the entire QS, N refers to the number of all clones. Genetic distance (D), number of synonymous substitutions per synonymous site (dS) and number of non-synonymous substitutions per non-synonymous site (dN) were calculated using MEGA 7.0 to quantify the diversity of HBV X QS. An online tool was used to analyse the mutations at the protein level (https://www.hiv.lanl.gov/content/sequence/ENTROPY/entropy.html).

### Cell culture

Huh 7 and Bel-7404 cell lines were maintained in Dulbecco’s modified Eagle’s medium (11995073, Gibco, USA) supplemented with the 10% foetal bovine serum (10099141, Gibco, USA) and incubated at 37°C in an atmosphere with 5% CO_2_.

### Construction of stable cell lines

To obtain HCC cell lines stably expressing wild-type and mutated HBx, Huh 7 and Bel-7404 cells were infected with the Lv-HBx-WT, Lv-HBx-L123S, Lv-HBx-S101P/L123S, Lv-HBx-T81P/S101P/L123S and lentivirus empty vector (GenePharma, China). The infection efficiency was confirmed using quantitative real time-PCR (qRT-PCR).

### Animal studies

Male BALB/c nude mice (4–5 weeks old) were purchased from Shanghai Lingchang Biological Technology Co., LTD, bred and maintained in a specific pathogen-free facility. For in vivo tumour growth assays, 1 × 10^7^ Huh 7 cells stably transfected with Lv-Vector, Lv-HBx, Lv-HBx-L123S, Lv-HBx-S101P/L123S or Lv-HBx-T81P/S101P/L123S in 100 μl phosphate-buffered solution were independently injected subcutaneously into the right-back of eight nude mice. All mice were sacrificed 28 days later. Mean tumour weight and volume for each group were measured.

### Cell counting kit-8 (CCK-8) assays, 5-ethynyl-2′-deoxyuridine (EdU) immunofluorescence staining, colony formation assays and cell cycle analysis

CCK-8 assays, EdU immunofluorescence staining, Colony formation assays and cell cycle analysis were performed as previously reported[25].

### Cell death analysis

Annexin V-PE and 7-Aminoactinomycin D (7-AAD) (559763, BD Biosciences, USA) were used to label dead cells, following the manufacturer's instructions. To detect the sensitivity of stable cell lines to genotoxic drugs, camptothecin (CPT) and dimethyl sulfoxide (DMSO) were used before fluorescence staining.

### Western blot analysis

After culturing stable HCC cell lines for 48 h, proteins were extracted using Cell lysis buffer for Western blot analysis (P0013J, Beyotime, China). Protein concentrations were measured using the BCA Protein Assay Kit (P0012S, Beyotime, China). Cell lysates were separated using 8% – 12% sodium dodecyl sulfate-polyacrylamide gel electrophoresis, transferred onto a polyvinylidene fluoride membrane (FFP24, Beyotime, China) and blotted with antibodies for Rb (#9309, CST, USA), pRb (##8516, CST, USA), E2F1 (ab288369, Abcam, UK), ATM (#2873, CST, USA), CDT1 (#8064, CST, USA), MYC (#5605, CST, USA), TOP1 (ab109374, Abcam, UK) and β-actin (#4970, CST, USA).

### Analysis for protein half-life

To detect the half-life of HBx-WT, HBx-SM, HBx-DM, HBx-TM and MYC, the plasmids expressing HBx-WT, HBx-SM, HBx-DM and HBx-TM were transiently transfected into Huh 7 cells. After 48 h, the cycloheximide (CHX) (HY-12320, MedChemExpress, USA) was added at a final concentration of 25 μg/mL and the protein levels of HBx-WT, HBx-SM, HBx-DM, HBx-TM and MYC were detected at certain time points by the western blot assays using antibodies for HBx (ab2741, Abcam, UK) and MYC (#5605, CST, USA).

### Co-immunoprecipitation assay

To analyse the interaction between HBx and MYC, the plasmids expressing HBx-WT, HBx-SM, HBx-DM and HBx-TM were transiently transfected into Huh 7 cells for 48 h. After lysis, HBx protein was immunoprecipitated by the Pierce Classic Magnetic IP/Co-IP Kit (88804, Thermo Fisher Scientific, USA) using the antibody (ab2741, Abcam, UK) against HBx and the protein levels of HBx and MYC were detected by the western blot assays using antibodies for HBx (ab2741, Abcam, UK) and MYC (#5605, CST, USA). Besides, the plasmid expressing MYC-Flag was also co-transfected with the plasmids expressing HBx-WT (pcHBx-WT), HBx-SM (pcHBx-SM), HBx-DM (pcHBx-DM) or HBx-TM (pc-HBx-TM) into Huh 7 cells for 48 h. After lysis, MYC-Flag was immunoprecipitated by the Pierce Classic Magnetic IP/Co-IP Kit using the antibody (#14793, CST, USA) against Flag tag and the protein levels of HBx and MYC-Flag were detected by the western blot assays using antibodies for HBx (ab2741, Abcam, UK) and Flag tag (#14793, CST, USA).

### Luciferase assays

*MYC* gene promoter regions (Upstream 2000bp to downstream 30 bp) were amplified from human genomic DNA, and the PCR products were cloned into promoter vector pGL3-Basic (Promega) to generate p*MYC*-pro plasmid. The plasmid p*MYC*-pro was co-transfected with pcHBx-WT, pcHBx-SM, pcHBx-DM or pcHBxTM into 293 T cells using Fugen HD (E2311, Promega, USA) according to the manufacturer's protocol. In addition, the pRL-TK vector, which expresses Renilla luciferase, was also co-transfected to correct the differences in both transfection and harvest efficiencies. Cell lysate was collected 48 h after transfection, and luciferase activities were measured using a Promega Dual-Luciferase Reporter Assay System. The activity of the firefly luciferase reporter was normalized to that of the Renilla luciferase.

### Ubiquitination assay

To dectect the ubiquitination level of MYC, the plasmid expressing MYC-Flag was also co-transfected with the plasmids expressing HBx-WT, HBx-SM, HBx-DM or HBx-TM into Huh 7 cells for 48 h. Then, the proteasome inhibitor MG-132 (HY-13259, MedChemExpress, USA) was added at a final concentration of 20 μM for 6 h before lysis, followed by co-IP and western blot analysis. The antibody (#3936, CST, USA) aginst the ubiquitin was used to detect the ubiquitination level of MYC.

### RNA extraction and qRT-PCR analysis

After culturing stable HCC cell lines for 24 h, total RNA was isolated from them using EasyPure RNA Kit (ER101, Transgen, China). Reverse-transcription was performed using Maxima H Minus cDNA Synthesis Master Mix (K1652, Thermo, USA). RT-qPCR was performed using TB Green Fast qPCR Mix (RR430, Takara, Japan). The relative transcript levels of target genes were normalized with glyceraldehyde 3-phosphate dehydrogenase (GAPDH) mRNA levels. The primers used for RT-qPCR were: MYC, (forward) 5′ – AGAGTTTCATCTGCGACCCG – 3′ and (reverse) 5′-GAGAAGCCGCTCCACATACA – 3′; GAPDH, (forward) 5′-GGAGCGAGATCCCTCCAAAAT-3′ and (reverse) 5′-CAGGAAACAGCTATGAC-3′.

### In silico prediction of the three-dimensional structures of HBx and MYC

The three-dimensional structures of wild-type HBx, mutated HBx and MYC were predicted and constructed in silico using I-TASSER[26] and PyMOL, respectively. The protein docking of HBx (wild-type or mutated) and MYC were performed in silico using Cluspro 2.0 and visualized using PyMOL.

### Karyotype analysis

The karyotype analysis for stable HCC cell lines was performed based on previous literature[27]. Briefly, 100 ug/mL colchicine was added to the culture medium, and the cells were incubated at 37°C for 70 min. Cells were washed and resuspended in 75 mM KCl for 18 min at 37°C. A total of 1.5 mL of 3:1 methanol:acetic acid was added dropwise to the resuspended cell pellets, followed by a 37°C water bath for 10 min. The cells were centrifuged and resuspended in 8 mL of 3:1 methanol:acetic acid solution. After centrifugation again, the supernatant was discarded, and 3:1 methanol:acetic acid solution was added to resuspend the cells. Following this, cells were dropped onto glass slides to obtain metaphase chromosome spreads. Spreads were stained with 4′,6-diamidino-2-phenylindole, and metaphase images were captured using a Nikon Eclipse microscope equipped with a CCD camera (Applied Spectral Imaging, Carlsbad, California) and 60× objective lens.

### Statistical analyses

Differences in measurement data were evaluated using paired or unpaired T-test and differences in enumeration data were evaluated using the chi-square test (Fisher's exact test was used when needed). IBM SPSS Statistics software (Version 22.0.0, IBM, USA) was used for the statistical analyses. All *P*-values were two-tailed, and *P *< 0.05 was considered statistically significant.

## Results

### Differential distribution of HBV X gene haplotypes and genotypes between intra-patient tumour and adjacent non-tumour tissues in patients with HCC

**Figure 1.**
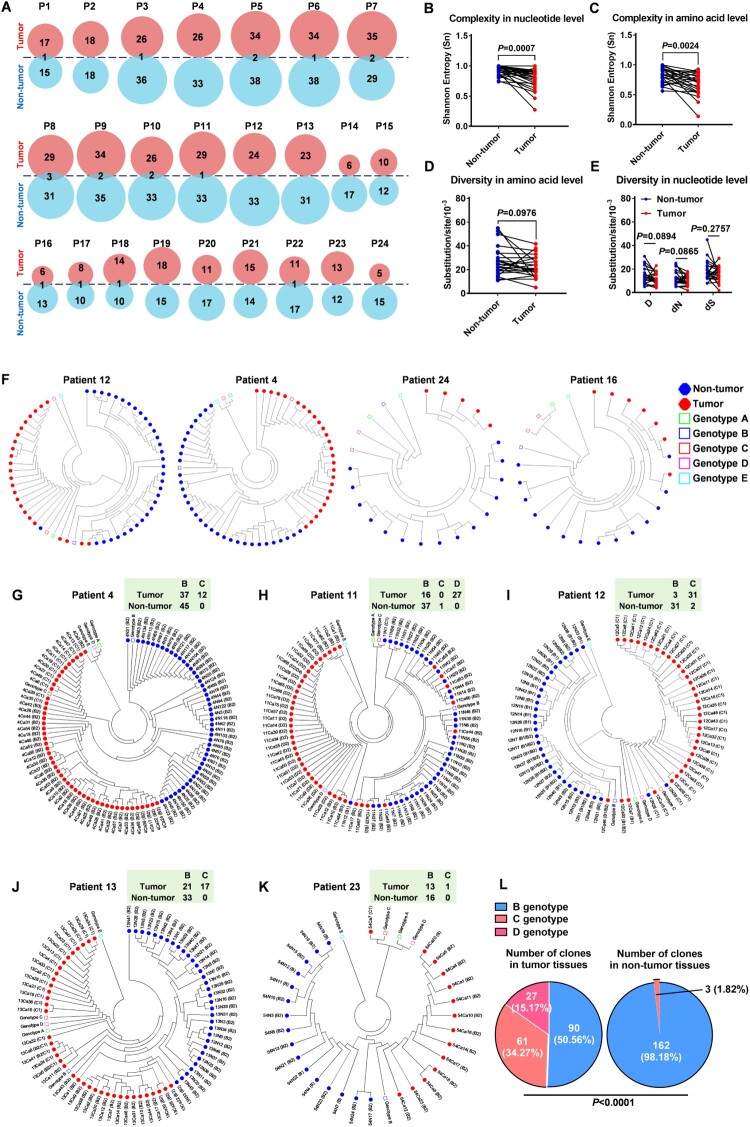
Differential distribution of HBV X gene haplotypes and genotypes exists between intra-patient tumour and adjacent non-tumour tissues in patients with HCC **(A)** Distributions of the haplotypes of the HBV X gene in the intra-patient tumour and non-tumour tissues. **(B-E)** Comparisons of the heterogeneity of HBV quasispecies (QS) across the X gene between tumour and non-tumour tissues. (B and C) The comparisons of the complexity quantified using Sn in the nucleotide (B) and amino acid level (C). (D and E) The comparisons of diversity were quantified using D at the amino acid level (D) and quantified using D, dS and dN at the nucleotide level (E). Sn, Shannon entropy. D, Genetic distance. dS, Number of synonymous substitutions per synonymous site. dN, number of non-synonymous substitutions per non-synonymous site. Each paired symbols represent the same patient's tumour tissue and adjacent non-tumour tissue; Paired t test; Paired n = 24. **(F)** Representative phylogenetic trees of HBV QS across the X gene from the liver tissue in patients with HCC. The solid red and blue dots indicate that the haplotypes are isolated from the tumour tissue and non-tumour tissue, respectively. The green, blue, red, purple and cyan hollow squares indicate the reference sequences for genotypes A, B, C, D and E, respectively. **(G-K)** All clones of the HBV X gene isolated from patients 4, 11, 12, 13 and 23 were genotyped using the phylogenetic trees. An online genotyping tool was used for the validation (https://www.ncbi.nlm.nih.gov/projects/genotyping/genotype.cgi). On the phylogenetic trees, the solid red and blue dots indicate that the clones are isolated from the tumour tissue and non-tumour tissue, respectively, and the green, blue, red, purple and cyan hollow squares indicate the reference sequences for genotypes A, B, C, D and E, respectively. **(L)** Numbers of genotyped clones with genotypes B, C and D were counted and compared between tumour and non-tumour tissues. The clones with the recombination of genotype B and C were found in patient 13 and counted as genotype B. Chi-square test was used to compare the difference of genotype distribution.

To investigate the characteristics of HBV QS across the X gene in the intra-patient tumour and adjacent non-tumour tissues, DNA cloning and sanger sequencing were performed to isolate the haplotypes of the HBV X gene in the postoperative liver tissues of 24 patients with HCC. The clinical characteristics and HBx protein levels in liver tissues of the 24 patients are shown in Table S1 and Figure S1. The patterns of HBV haplotypes across the X gene reflected that the characteristics of HBV QS across the X gene were different between the tumour and non-tumour tissues in a certain patient with HCC, and the predominant haplotypes were found in the tumour tissues of most patients with HCC (Figure S2), indicating a difference in the heterogeneity of HBV QS across the X gene between tumour and non-tumour tissues. Further, the distributions of haplotypes in the intra-patient tumour and non-tumour tissues were analysed. The different haplotypes of the X gene varied between tumour and adjacent non-tumour tissues in all 24 patients (Figure 1A), suggesting that the evolution of the HBV X gene was independent. To confirm the difference in the heterogeneity of HBV QS across the X gene between tumour and non-tumour tissues, the heterogeneity was quantified into two aspects: complexity and diversity. The complexity of HBV QS across the X gene in tumour tissues was observed to be lower than that in non-tumour tissues at both aa and nucleotide levels (Figures 1B and C). Despite the lack of significant differences owing to the limited sample size, the diversity of HBV QS across the X gene in tumour tissues was lower than that in non-tumour tissues at both aa and nucleotide levels (Figures 1D and E). Besides, the difference in the heterogeneity of HBV QS across the X gene between different genders was also analysed. Although the number of female cases was limited, the heterogeneity of HBV QS across the X gene in female patients seemed to be lower than that in male patients (Figure S3), which needed to be confirmed with more cases in the future. Additionally, to confirm the difference in the evolution of the HBV X gene between intra-patient tumour and non-tumour tissues, the phylogenetic analysis for the haplotypes of the HBV X gene in the liver tissue of each patient with HCC was performed. The phylogenetic distribution of the haplotypes of the HBV X gene showed a polarized propensity according to the different tissue sources in most patients with HCC (Figure 1F and Figure S4). Therefore, the evolution of the HBV X gene is distinct between the intra-patient tumour and adjacent non-tumour tissues in patients with HCC.

**Figure 2.**
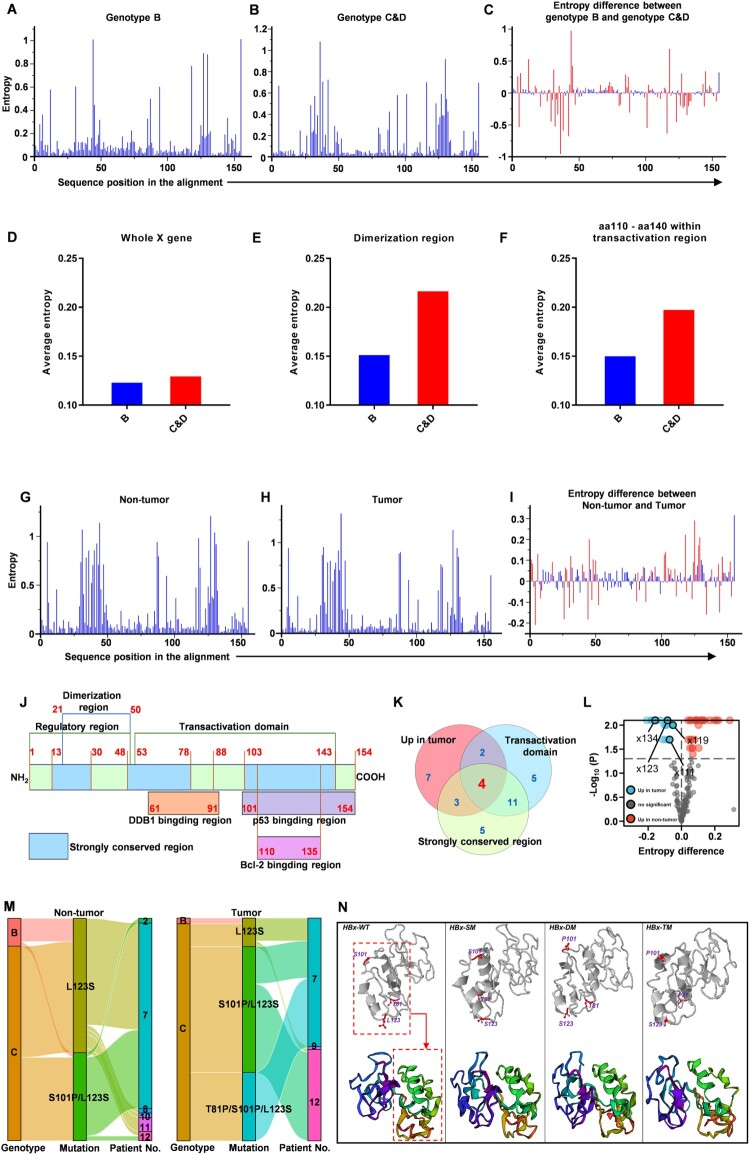
L123S mutation increases and exists in linkage with S101P or S101P plus T81P in the liver tumour tissues **(A-C)** Mutations at each amino acid site across the X gene were assessed using Shannon entropy (Sn). An online tool was used to calculate and illustrate Sn (https://www.hiv.lanl.gov/content/sequence/ENTROPY/entropy.html). (A) Sn at each amino acid site in genotype-B clones. (b) Sn at each amino acid site in genotype-C and genotype-D clones. (C) Differences in Sn at each amino acid site between genotype-B and genotype-(C&D) clones; Significant sites with *P*≤0.05 are shown in red bars. **(D-F)** Average mutation frequency across different fragments within the X gene was assessed via average entropy. (D) Average entropy across the whole X gene. (E) Average entropy of dimerization region from amino acid 21 to amino acid 50. (F) Average entropy between amino acid 110 to amino acid140 within the transactivation region. **(G-I)** Shannon entropy (Sn) at each amino acid site was used to analyse the mutations across the HBV X gene for the clones isolated from the tumour tissues or non-tumour tissues. (G) Sn at each amino acid site in non-tumour tissues (n = 680). (H) Sn at each amino acid site in tumour tissues (n = 711). (I) Differences in Sn at each amino acid site between non-tumour and tumour tissues; Significant sites with *P*≤0.05 are shown in red bars. **(J)** A schematic diagram of the functional domains of HBV X protein. **(K)** The number of the significant sites which have higher mutation frequencies, located within the transactivation domain or strongly conserved region are displayed in the Venn diagram. **(L)** Significant sites, including x111, x119, x123 and x134, with higher mutation frequencies, located within the transactivation domain and strongly conserved region simultaneously are displayed in the volcano plot. **(M)** Clones of HBV X genes harbouring mutation L123S, double mutation S101P/L123S and triple mutation T81P/S101P/L123S are displayed in the Sankey diagram. **(N)** Three-dimensional structures of wild-type and mutant HBx proteins. WT, wild type. SM, single mutation, refers to L123S. DM, double mutation, refers to S101P/L123S. TM, triple mutation, refers to T81P/S101P/L123S.

Given that the evolutions of HBV QS across the X gene are distinct between the intra-patient tumour and adjacent non-tumour tissues in patients with HCC, this study further aimed to determine if different genotypes could also have a different distribution trend between tumour and non-tumour tissues. As HBV genotypes C and D are more strongly associated with the development of HCC than genotypes A and B in the CHB population[28], genotypes C and D were hypothesized to show an upward trend in progression in tumour tissues compared with genotypes A and B at the tissue level. Additionally, 5 of 24 patients with HCC in this study were found to be co-infected with HBV of multiple genotypes based on the HBV X gene (Figure 1G-K), which made it possible to investigate the distribution trend of different HBV genotypes in the liver tissues. Among these five patients, one (patient 11) was co-infected with genotypes B, C and D (Figure 1H), while the others (patients 4, 12, 13 and 23) were co-infected with genotypes B and C (Figure 1G, I-K). On counting the number of the genotyped clones of the HBV X gene, patients 4, 12, 13 and 23 showed that the clones of B genotype versus (vs) C genotype in the tumour tissues were 37 vs 12, 3 vs 31, 21 vs 17 and 13 vs 1, respectively, while the clones of B genotype vs C genotype in the non-tumour tissues were 45 vs 0, 31 vs 2, 33 vs 0 and 16 vs 0, respectively (Figure 1G, I-K). Moreover, in patient 11, the clones of B genotype vs C genotype vs D genotype in the tumour tissue was 16 vs 0 vs 27, while the clones of B genotype vs C genotype vs D genotype in the non-tumour tissue was 37 vs 1 vs 0 (Figure 1H). In total, in these 5 patients, the number of clones of genotypes C and D in the tumour tissues were more than that in the non-tumour tissues (88/90 vs 3/162, *P *< 0.0001) (Figure 2F). Therefore, the HBV strains of genotypes C or D prefer to localize in the tumour tissues compared with those of genotype B in patients with HCC co-infected with multiple HBV genotypes.

### A novel combination of mutation at aa residues 81, 101 and 123 of HBx protein arises in the liver tumour tissues

Based on the above finding, the varied distribution between genotypes B, C and D in the liver tumour and non-tumour tissues was speculated to be attributed to the difference in mutation spectrums across the X gene among the genotypes. Therefore, the mutations between the genotype B and genotype (C&D) clones of the X gene were preliminarily analysed and compared by calculating the Sn of each aa site. Compared with genotype B clones (Figure 2A), the mutant hot spots in genotype (C&D) clones exhibited two clusters, including the dimerization region ranging from aa21 to aa50 and the fragment ranging from aa110 to aa140 within the transactivation region (Figure 2B and J), which was consistent with the entropy difference between genotype B and genotype (C&D) (Figure 2C). To further validate the results, the average entropies of the whole X gene, dimerization region and fragment from aa110 to aa140 in genotype B and genotype (C&D) clones were calculated (Figure 2D-F). The average entropy of the whole X gene in genotype (C&D) clones was slightly higher than in genotype B clones (Figure 2D). However, the average entropies of the dimerization region and fragment from aa110 to aa140 within the transactivation region in genotype (C&D) clones were much higher than those in genotype B clones (Figure 2E-F). Given that the dimerization region and transactivation region of HBx protein was reported to be closely associated with HBx function[4], the mutation spectrum across the X gene in genotype C and D likely alters the function of HBx protein compared with genotype B.

The mutations across the X gene are also likely to be compartmentalized between tumour and non-tumour tissues. To verify this, Sn of each aa site across the X gene in the 680 and 711 clones isolated from the non-tumour and tumour tissues, respectively, was calculated, and the entropy difference between non-tumour and tumour tissues was compared (Figure 2G-I). Furthermore, aa sites with significant differences formed mainly two clusters ranging roughly from aa site 25 to aa site 50 and from aa site 100 to site 140 (Figure 2I). More precisely, 44 aa sites were showing significant entropy differences between tumour and non-tumour tissues, including 16 and 28 aa sites with higher Sn in the tumour and non-tumour tissues, respectively (Figure 2I and Table S3). Further, a schematic diagram of the functional domains of HBV X protein was drawn to identify mutations influencing HBx protein function according to previous literature[4] (Figure 2J). Apart from the 16 aa sites with higher Sn in the tumour tissues, there were 22 and 23 significant aa sites located within the transactivation domain and the strongly conserved region, respectively, of which four aa sites overlapped among these three mutation types (Figure 2K), including x111, x119, x123 and x134 (Figure 2L). Among these four aa sites, mutation L123S was the major residue change at x123 (Table S2), and it co-existed mostly with S101P or S101P plus T81P in the tumour tissues while the clones of the HBV X gene harbouring the single-point mutation L123S and double mutation S101P/L123S were the major mutants in the non-tumour tissues (Figure 2M and Figure S3). Since the T81P, S101P and L123S mutations were all within the transactivation domain, the triple-point mutation T81P/S101P/L123S was likely to change the conformation of HBx protein. To validate this hypothesis, three-dimensional structures for HBx proteins, including the wild type (denoted as WT), L123S mutant (denoted as SM), S101P/L123S mutant (denoted as DM) and T81P/S101P/L123S mutant (denoted as TM), was constructed in silico (Figure 2N). The conformation of HBx-TM protein had a significant change (Figure 2N), implying a potential functional change, compared to HBx-WT. In addition, to vestigate the effect of conformational change on the half-life of HBx, the half-lifes of HBx-WT, HBx-SM, HBx-DM and HBx-TM were also analysed by the western blot assays, displaying that HBx possessing double (S101P/L123S) or triple mutations (T81P/S101P/L123S) had a longer half-life compared with wild-type HBx and HBx with the single mutation (L123S) (Figure S6). In total, the HBx mutants with triple mutation T81P/S101P/L123s arise more frequently in the liver tumour tissues, indicating their potential role in HCC progression.

### HBx protein harbouring triple mutation T81P/S101P/L123S can elevate the MYC level compared with wild-type HBx

**Figure 3.**
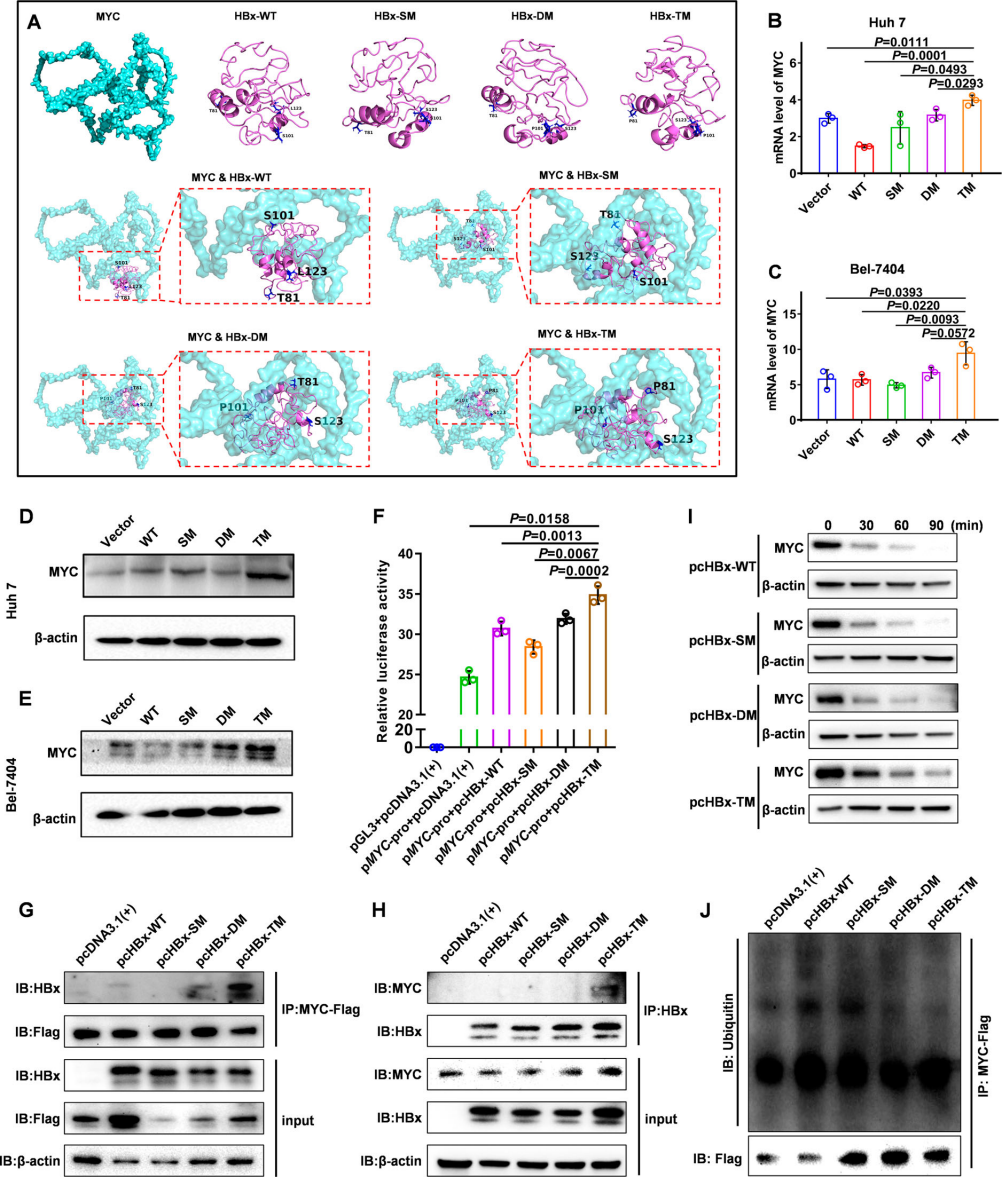
HBx-T81P/S101P/L123S upregulates the level of MYC compared with wild-type HBx **(A)** Protein dockings between wild-type HBx and MYC or between mutant HBx and MYC were predicted in silico. Three dimensional structures of wild-type HBx, mutant HBx and MYC are displayed respectively at the top of the panel. Three dimensional structures of docking between wild-type HBx and MYC or between mutant HBx and MYC are displayed in the middle and bottom of the panel. **(B and C)** The mRNA expressions of MYC in Huh 7 (B) and Bel-7404 (C) were assessed using a quantitative polymerase chain reaction. Each symbol represents an individual experiment; Data are presented as mean ± standard error of the mean; Unpaired t test; n = 3. **(D and E)** Protein levels of MYC in hepatocellular carcinoma cells. Western blot was used to assess the protein level of MYC in the Vector, WT, SM, DM and TM groups in Huh 7 (D) and Bel-7404 (E) cells, respectively. β-actin was used as a loading control. **(F)** The transcriptional activity of *MYC* promoter was assessed by the luciferase assays in 293T cells after the plasmid p*MYC*-promoter was co-transfected with pcHBx-WT, pcHBx-SM, pcHBx-DM or pcHBx-TM into 293T cells; Data are presented as mean ± standard error of the mean; Unpaired t test; n = 3. **(G and H)** The interactions between wild – or mutant-type HBx and MYC were assessed by the co-immunoprecipitation assays. After MYC-Flag (G) or HBx (H) was immunoprecipitated, the western blot assays were performed to detect the protein levels of HBx (G) and MYC (H) in Huh 7 cells. **(I)** The western blot assays were used to analyse the change of MYC protein level at 0th, 30th, 60th and 90th minute after the treatment of cycloheximide (CHX) at a final concentration of 25 μg/mL in the Huh 7 cells transiently expressing HBx-WT, HBx-SM, HBx-DM or HBx-TM. **(J)** The western blot assays were used to assess the ubiquitination levels of MYC by the antibody aginst the ubiquitin after the MYC-Flag was immunoprecipitated in the Huh 7 cells transiently expressing HBx-WT, HBx-SM, HBx-DM or HBx-TM.

Since HBx was reported to interact with MYC and participate in the development of HCC[29–31], the influence of wild-type and mutant HBx on the level of MYC was investigated in this study. The three-dimensional conformations for HBx-WT, HBx-SM, HBx-DM, HBx-TM and MYC were predicted and constructed using I-TASSER and PyMOL, respectively. Then, protein docking between wild-type HBx and MYC or between mutant HBx and MYC was predicted and constructed using Cluspro 2.0 and PyMOL, respectively. Moreover, mutation L123S altered the site of protein docking between HBx-WT and MYC (Figure 3A). Furthermore, as the number of mutations increased, the docking position between the mutant HBx and MYC had a trend to be tighter (Figure 3A), suggesting that HBx-TM could impact the expression or function of MYC. To verify this speculation, the mRNA and protein levels of MYC in HCC cell lines stably expressing wild-type HBx, mutant HBx and lentiviral empty vector were analysed using quantitative PCR (qPCR) and western blot assay, respectively. Compared with HBx-WT, HBx-TM significantly elevated the mRNA and protein levels of MYC in Huh7 and Bel-7404 (Figure 3B-E). Further, to verify that HBx-TM could elevate MYC expression at the transcriptional level, the plamid pGL3 with the promoter region of *MYC* gene (p*MYC*-promoter) was co-transfected with the plasimid pcDNA3.1(+) expressing HBx-WT (pcHBx-WT), HBx-SM (pcHBx-SM), HBx-DM (pcHBx-DM) or HBx-TM (pcHBx-TM) respectively in the 293 T cells and then the luciferase assays were performed. The luciferase activity was significantly higher in the 293 T cells expressing HBx-TM than that in the 293 T cells expressing HBx-WT (Figure 3F). To confirm the enhanced interaction between HBx-TM and MYC, the co-immunoprecipitation (Co-IP) assays were performed. The results showed that HBx-TM indeed had a higher targeting potential to MYC compared with other types of HBx in this study (Figure 3G and H). Considering that the enhanced interaction between HBx-TM and MYC might have a effect on the turnover of MYC protein at the post-translation level, the half-life of MYC was analysed. Compared with other types of HBx in this study, HBx-TM could prolong the half-life of MYC protein (Figure 3I). Moreover, the ubiquitination level of MYC protein was lower in the Huh 7 cells expressing HBx-TM than that in the Huh 7 cells expressing HBx-WT (Figure 3J). Hence, the HBx protein harbouring triple mutation T81P/S101P/L123S can elevate the level of MYC via the transcription and post-translation processes compared with wild-type HBx, indicating an underlying tumour-promoting effect of HBx-T81P/S101P/L123S.

### Triple mutation T81P/S101P/L123S confers a tumour-promoting phenotype on HBx

**Figure 4.**
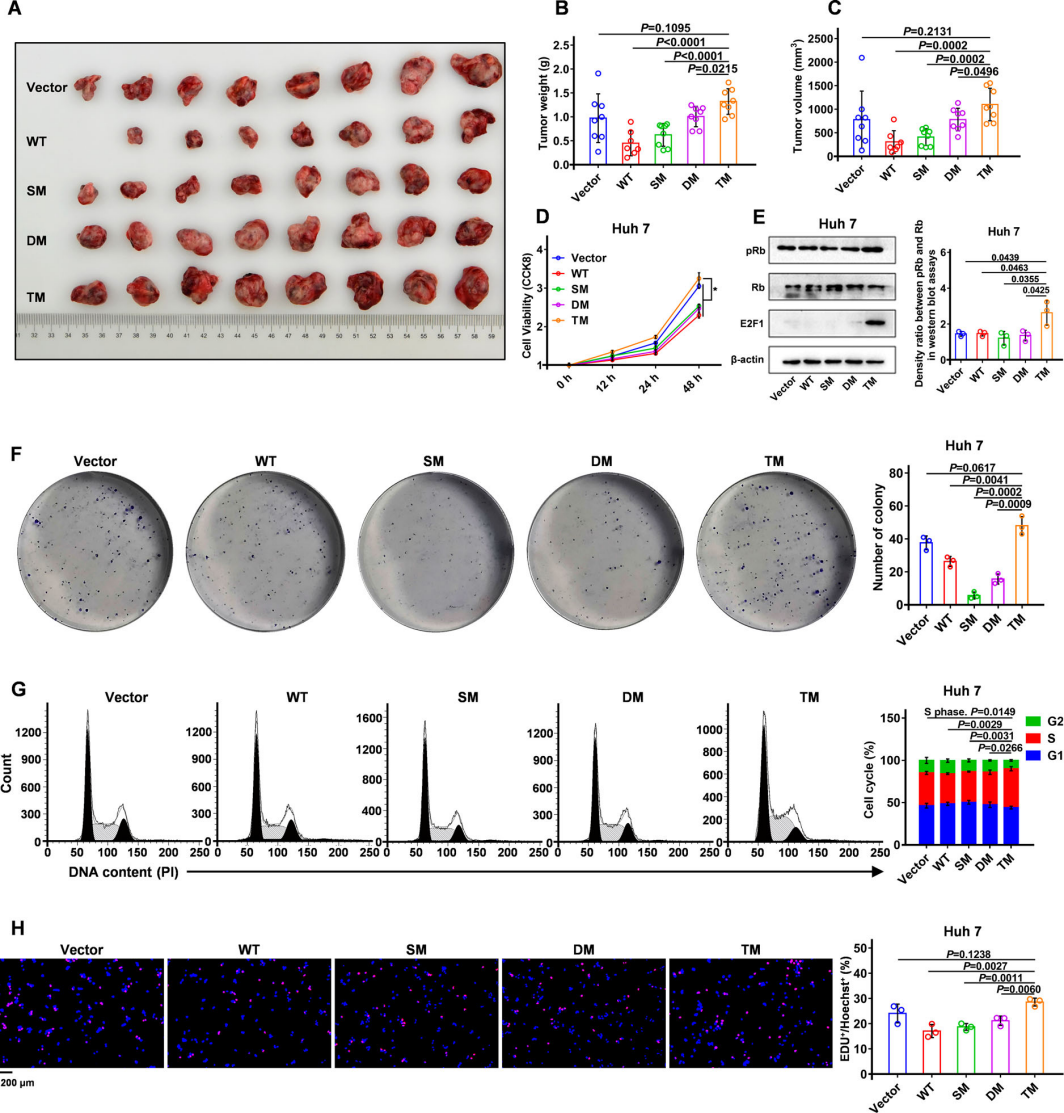
HBx-T81P/S101P/L123S promotes the growth and proliferation of hepatocellular carcinoma (HCC) cells compared with wild-type HBx **(A)** Tumour masses harvested from the nude mice injected subcutaneously with Huh 7 cells. A total of 1 × 10^7^ Huh 7 cells stably transfected with Lv-Vector, Lv-HBx-WT, Lv-HBx-SM, Lv-HBx-DM or Lv-TM in 100 μl phosphate buffer solution was independently injected subcutaneously into the right-back of eight nude mice. **(B and C)** Weight (B) and volume (C) of tumour masses were assessed on the 28th day after the inoculation. Data are presented as mean ± standard error of mean (SEM); Unpaired t test; Each symbol represents an individual tumour tissue; n = 8 for the Vector, SM, DM and TM group; n = 7 for the WT group. **(D)** Cell viabilities of Huh 7 cells were assessed by cell counting kit-8 (CCK-8) assays. A total of 1 × 10^4^ Huh 7 cells were seeded and cultured for 12 h in each well of a 96-well plate. Subsequently, CCK-8 assays were performed at four time points (0, 12, 24, 48 h) among the Vector, WT, SM, DM and TM groups; Data are presented as mean ± SEM. Unpaired t-test; n = 6. **(E)** Protein levels of E2F1, Rb and pRb in Huh 7 cells were assessed using western blot. β-actin was used as a loading control. E2F1, E2F Transcription Factor 1. Rb, RB Transcriptional Corepressor 1. pRb, phosphorylated Rb. **(F)** Colony formation assays were performed to evaluate the single-cell growth ability in Huh 7 cells. Differences in colony numbers among the Vector, WT, SM, DM and TM groups are presented on the right of the panel; Data are presented as mean ± SEM; Unpaired t test; n = 3. **(G)** Cell cycles were analysed using flow cytometry of Huh 7 cells. Propidium iodide (PI) was used for the detection of DNA contents. G1 phase, first growth phase or post mitotic gap phase. S phase, DNA replication phase. G2 phase, cell growth phase. Proportions of cells in the S phase in the Vector, WT, SM, DM and TM groups are presented at the right of the panel; Each symbol represents an individual experiment. Data are presented as mean ± SEM; Unpaired t test; n = 3. **(H)** Levels of DNA replication were examined using 5-ethynyl-2'-deoxyuridine (EdU) incorporation assays in Huh 7 cells. EdU with Alexa Fluor 555 showing red fluorescence was used to label the DNAs replicating. The Hoechst stain with blue fluorescence was used to label the general DNAs. The proportions of cells with EdU staining in the Vector, WT, SM, DM and TM groups are presented at the right of the panel; Each symbol represents an individual experiment; Data are presented as mean ± SEM; Unpaired t test. n = 3; Original magnification, ×400.

To confirm the underlying tumour-promoting effect of HBx-T81P/S101P/L123S, the lentiviral vector was used to obtain the Huh 7 cell lines stably expressing the wild-type HBx, mutant HBx and lentiviral empty vector, namely Huh 7-WT, Huh 7-SM, Huh 7-DM, Huh 7-TM and Huh 7-Vector. The Huh 7 cell lines were subcutaneously injected into the nude mice for the construction of the xenograft tumour models. Then, the tumour masses harvested from the nude mice injected subcutaneously with Huh 7-TM cells were found to have a larger mean weight and volume than those harvested from the nude mice injected subcutaneously with Huh 7-WT cells (Figure 4A-C and Figure S7). Subsequently, the cell viabilities of Huh 7 cells were assessed using CCK-8 assays in vitro, wherein the cell viability of Hun 7-TM cells was greater than that of Huh 7-WT (Figure 4D). Moreover, the cell colony formation assays evaluating the growth ability of the single tumour cell in Huh 7 cells showed greater growth ability of the single Huh 7-TM cell than the single Huh 7-WT cell (Figure 4F). These in vivo and in vitro results indicate that HBx-T81P/S101P/L123S could promote the growth and proliferation of Huh 7 cells compared with wild-type HBx. Accurate G1/S transition is crucial for the control of cell proliferation and its misregulation promotes oncogenesis. E2F Transcription Factor 1 (E2F1), RB Transcriptional Corepressor 1 (Rb) and phosphorylated Rb (pRb) are involved in the regulation of the G1/S transition and were assessed using Western blot assays. The Huh 7-TM cells were found to have higher protein levels of E2F1 and pRb than the Huh 7-WT cells (Figure 4E), indicating that more Huh 7-TM cells were likely to enter the S phase compared with the Huh 7-WT cells. Notably, the density ratio between pRb and Rb was not parallel to the E2F1 protein level, indicating that there might be other Rb-independent mechanisms for the regulation of E2F1. In fact, Li et al. [32] have reported that MYC stabilizes the mRNA of E2F1 by activating the lncRNA USP2-AS1, thereby increasing the level of E2F1. Hence, the elevated MYC level by HBx-T81P/S101P/L123S (Figure 3D and E) might also be involved in the regulation of E2F1 levels. To verify this suggestion, propidium iodide (PI) was used to stain DNAs and then the cell cycles were analysed using flow cytometry in Huh 7 cells. A significantly higher number of Huh 7-TM cells were found in the S phase compared with the Huh 7-WT cells (Figure 4G). As DNA synthesis commences when cells enter the S phase, EdU was used to label the DNAs replicating while Hoechst was used to label the general DNAs to further assess the enhanced DNA replication of the Huh 7-TM cells compared with the Huh 7-WT cells. A higher number of Huh 7-TM cells were found to be labelled with Edu than Huh 7-WT cells (Figure 4H), revealing that there were more replicating cells in the Huh 7-TM cells than in the Huh 7-WT cells. Similar results were also found in the Bel-7404 cells in vitro (Figure S8). Therefore, compared with wild-type HBx, HBx-T81P/S101P/L123S can promote the growth and proliferation of HCC cells by regulating the G1/S transition.

Since excessive or continuous DNA replication in cancers is reported to lead to a higher demand for DNA repair components, which increases the risk of the genome instability[33], HBx-T81P/S101P/L123S was speculated to increase the genomic instability of the HCC cells compared with wild-type HBx. Furthermore, DNA damage shows increased sensitivity to genotoxic drugs, such as the CPT [34], hence, annexin V-PE and 7-AAD were used to label the dead cells in the presence of CPT or DMSO. The results showed that in the presence of DMSO, no difference was observed in the proportion of cells labelled with annexin V-PE among the HCC lines stably expressing HBx-WT, HBx-SM, HBx-DM, HBx-TM and lentiviral empty vector (Figures S9A and S9C). However, in the presence of CPT, the proportion of cells labelled with annexin V-PE in the HCC cell lines stably expressing HBx-TM was significantly higher than in the HCC cell lines stably expressing HBx-WT (Figures S9B and S9D), indicating that HBx-T81P/S101P/L123S is likely to increase the genomic instability in HCC cells compared with wild-type HBx. Ataxia telangiectasia mutated protein kinase (ATM) is an important cell cycle checkpoint kinase, which senses double-stranded breaks (DSB) for maintaining genome stability[35]. Chromatin licensing and DNA replication factor 1 (CDT1) are also involved in the regulation of DNA replication, and the overexpression of CDT1 could lead to genome instability[36]. Therefore, the protein levels of CDT1 and ATM in HCC cells were assessed using Western blot assays, wherein the HCC cells stably expressing HBx-TM had higher CDT1 and ATM protein levels compared with the HCC cells stably expressing HBx-WT (Figures S9E and S9F), suggesting that the presence of DNA DSB in the HCC cell lines stably expressing HBx-TM. To verify this suggestion, karyotype analysis was performed in the HCC cells stably expressing wild-type HBx, mutant HBx and lentiviral empty vector. Broken chromosomes were found in the HCC cell lines stably expressing HBx-TM (Figures S9G and S9H), which was consistent with the results of the Western blot assays (Figures S9E and S9F). Therefore, HBx-T81P/S101P/L123S has a proliferation-enhancing effect compared with wild-type HBx and can increase the genomic instability of HCC cells, which is associated with the development of HCC.

### MYC participates in the tumour-promoting effect of HBx protein conferred by the triple mutation T81P/S101P/L123S

**Figure 5.**
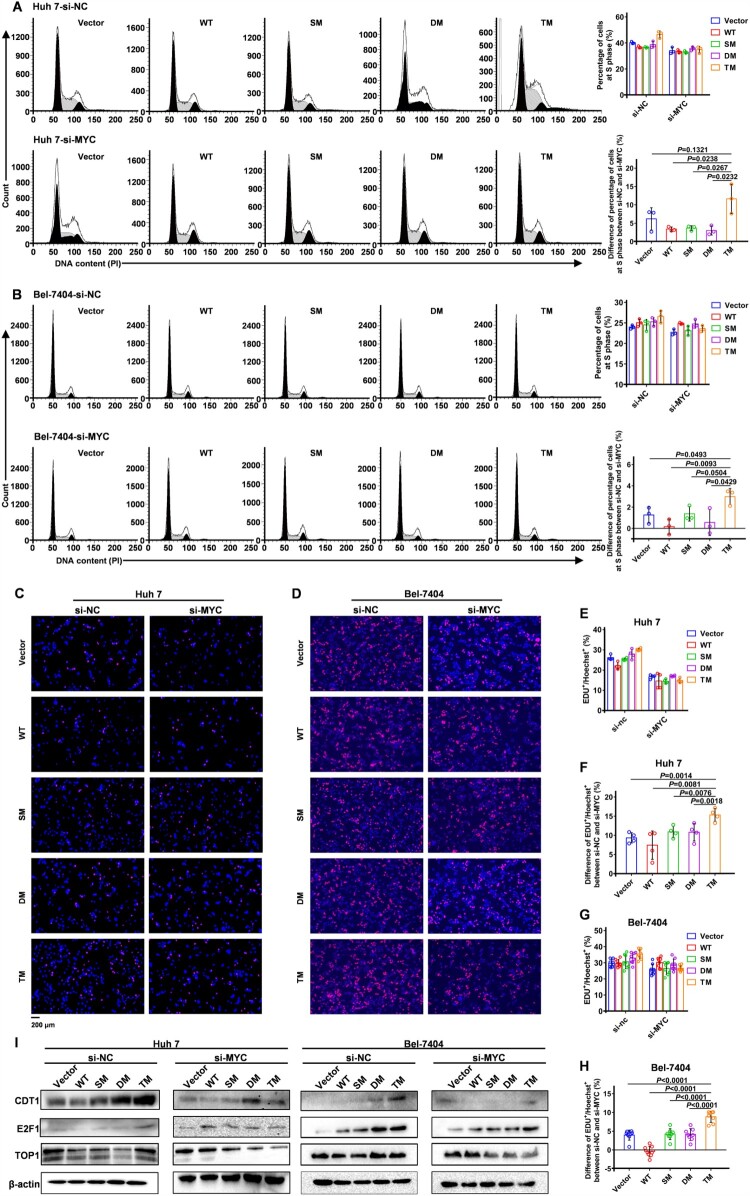
Knockdown of MYC can abrogate the tumour-promoting effect of HBx conferred by mutation T81P/S101P/L123S **(A and B)** Cell cycles were assessed using flow cytometry in Huh 7 cells (A) and Bel-7404 cells (B). The siRNA (10 pmol) of MYC or the control was transfected into Huh 7 cells and Bel-7404 cells in a 6-well dish, respectively. The ratios of cells in the S phase among the Vector, WT, SM, DM and TM groups are presented on the right at the top of the panel in (A) or (B); Each symbol represents an individual experiment. The differences in the ratios of cells in the S phase between cells transfected with si-MYC and with si-NC are presented on the right at the bottom of the panel in (A) or (B); Each symbol represents a difference in the ratios of cells in the S phase between a certain cell transfected with si-MYC and a matched cell transfected with si-NC. Data on the right of the panel in (A and B) are presented as mean ± standard error of mean (SEM); Unpaired t test; n = 3. **(C-H)** Levels of DNA replication were examined by the EdU incorporation assays in HCC cells. The siRNA (10 pmol) of MYC or the control was transfected into Huh 7 cells and Bel-7404 cells in a 6-well dish respectively. (C and D) the representative figures reflecting the replicating cells labelled by EdU in Huh 7 cells (C) and Bel-7404 cells (D) are displayed. The nuclei labelled by EdU in the replicating cells show the red fluorescence. The general nuclei labelled using Hoechst show the blue fluorescence. Original magnification, ×400. (E and G) The ratios of cells labelled by EdU among the Vector, WT, SM, DM and TM groups in Huh 7 cells (E) and Bel-7404 cells (G) are presented. Each symbol represents an individual experiment. (F and H) The differences in the ratios of cells labelled by EdU in Huh 7 cells (F) and Bel-7404 cells (H) between cells transfected with si-MYC and with si-NC are presented. Each symbol represents a difference in the ratios of cells labelled by EdU between a certain cell transfected with si-MYC and a matched cell transfected with si-NC. Data in (E-H) are presented as mean ± SEM; Unpaired t test; n = 4 in Huh 7 cells; n = 8 in Bel-7404 cells. **(I)** Protein levels of CDT1, E2F1 and TOP1 in HCC cells were assessed by the western blot among the Vector, WT, SM, DM and TM groups. The siRNA (10 pmol) of MYC or the control was transfected into Huh 7 cells (left of the panel) and Bel-7404 cells (right of the panel) in a 6-well dish respectively. β-actin was used as a loading control. TOP1, DNA Topoisomerase I.

To determine if the proliferation-enhancing effect of HBx-T81P/S101P/L123S was related to the upregulation of MYC, small interfering RNA (siRNA) targeting MYC (si-MYC) and its negative control siRNA (si-NC) were respectively transfected into the Huh 7 cells and Bel-7404 cells stably expressing HBx-WT, HBx-SM, HBx-DM, HBx-TM and lentiviral empty vector. Then, the cell cycles were analysed using flow cytometry. In the control group (hereinafter referred to as the si-NC group), the number of cells in the S phase in the Huh 7-TM/Bel-7404-TM cells was more than those in the Huh 7-WT/Bel-7404-WT cells (Figure 5A and B), which was consistent with the results of cell cycle detection in Figure 4G and Figure S8B. Nevertheless, when MYC was downregulated by si-MYC (hereinafter referred to as the si-MYC group), no difference in cell numbers in the S phase was found between Huh 7-WT/Bel-7404-WT and Huh 7-TM/Bel-7404-TM cells (Figure 5A and B), indicating that the role of promoting the G1/S transition was dependent on the expression of MYC. Subsequently, the levels of DNA replication were further evaluated and compared using EdU incorporation assays in the Huh 7/Bel-7404-Vector, Huh 7/Bel-7404-WT, Huh 7/Bel-7404-SM, Huh 7/Bel-7404-DM and Huh 7/Bel-7404-TM cells between the si-NC and si-MYC groups. As shown in Figure 4H and Figure S8C, the ratios of replicating cells labelled by EdU in Huh 7/Bel-7404-TM cells were higher than the Huh 7/Bel-7404-WT cells in the si-NC group (Figure 5C,D, E and G). However, in the si-MYC group, no difference in the ratios of the replicating cells was observed between Huh 7-WT/Bel-7404-WT and Huh 7-TM/Bel-7404-TM cells (Figure 5C–H), which further highlighted the role of MYC in the tumour-promoting effect of HBx conferred by mutation T81P/S101P/L123S. Additionally, the protein levels of DNA Topoisomerase I (TOP1), whose overexpression is related to the genome instability, EDT1 and E2F1 were detected using Western blot assays. In the si-NC group, the protein levels of TOP1, CDT1 and E2F1 in Huh 7/Bel-7404-TM cells were higher than those in Huh 7/Bel-7404-WT cells (Figure 5I). However, this difference between Huh 7/Bel-7404-WT and Huh 7/Bel-7404-TM cells vanished in the si-MYC group (Figure 5I). Therefore, the tumour-promoting effect conferred by the triple mutation T81P/S101P/L123S on the HBx protein is dependent on the expression of MYC.

## Discussion

This study provides a comprehensive analysis of the characteristics of HBV haplotypes across the X gene in the liver microenvironment in patients with HCC. The evolution of the HBV X gene was demonstrated to be compartmentalized between intra-patient tumour and non-tumour tissues in HBV-related patients with HCC. Further, genotypes C and D were associated with the development of HCC compared with genotype B in the CHB population. Moreover, at the tissue level, genotypes C and D are localized in the tumour tissues compared with genotype B. Additionally, some mutations across the X gene could potentially affect HBx protein function. Importantly, HBx with triple mutation T81P/S101P/L123S possesses a tumour-promoting effect compared with wild-type HBx. These findings aid in improving our knowledge of the pathogenesis of HBV-related HCC. The current study provides three main insights into HBV X genotype-phenotype characteristics during HCC development.

First, the evolution of the HBV X gene in tumour tissues was strikingly different from that in non-tumour tissues, which could be attributed to its QS nature with a spectrum of viral haplotypes harbouring mutations conferring distinct fitness to certain environments in individuals infected with HBV[7, 8]. Haplotypes adapting to the change in the liver microenvironment are selected and gradually become predominant. Therefore, variations in HBV QS reflect changes in the internal microenvironment of infected cells. Considerable studies on the characteristics of HBV QS in HBV-related liver diseases have been reported[10–13]; however, these studies were based on peripheral blood. Considering that the liver is the only organ known to support HBV replication, studies on the characteristics of HBV QS in the liver during the progression of HBV-related liver diseases could better reflect the changes in the liver microenvironment. Studies on the differences in the characteristics of HBV QS across PreS and core promoter regions between tumour and non-tumour tissues are not scarce. This study complements our understanding of the characteristics of HBV QS across the X gene in the liver microenvironment of patients with HCC. The haplotypes across the HBV X gene in the tumour tissues were found to have little overlap with those in the non-tumour tissues, and the evolution of the HBV X gene showed compartmentalization between the intra-patient tumour and non-tumour tissue in most patients with HCC. Moreover, the heterogeneity of haplotypes across the X gene in tumour tissues, especially its complexity, was significantly lower than that in adjacent non-tumour tissues, which could be attributed to the presence of predominant haplotypes in tumour tissues. Accordingly, these predominant haplotypes were speculated to be localized in the liver tumour microenvironment and potentially conducive to the formation and maintenance of the liver tumour microenvironment.

Second, an explanation is provided for the clinical phenomenon wherein patients infected with genotype C or D HBV have a higher risk of HCC development compared with patients infected with genotype B HBV. It is well-known that genotypes B and C are most prevalent in Asia and Oceania [16]. Moreover, genotypes C and D are closely associated with the development of HCC[16, 17]. Consistent with the association between genotypes and the progression of HBV-related liver diseases to HCC in the CHB population, this study provides evidence at the tissue level that genotypes C and D have a bias to localize in the tumour tissues compared with genotype B in patients with HCC co-infected with multiple-genotype HBV. Nevertheless, the mechanism underlying the infection with genotype C or D and the increasing risk of HCC remains unknown. High serum HBV DNA levels are correlated in the clinical setting with liver damage accumulation, evolution to cirrhosis and development of HCC[37]. Malmström et al.[38] detected HBV loadings in 124 patients with CHB who were followed up for a median of 9.2 years, revealing that patients with genotype C or D who had high loadings at baseline still maintained higher viral loadings compared with those with genotypes A and B at the time of follow-up. Further, the higher HBV loading in patients with genotype C or D was considered a risk factor for HCC development. However, Malmström et al.[38] included patients who received the antiviral treatment of interferon (IFN) or lamivudine (LMV). In fact, patients with genotype C had a relatively poorer response to IFN than those with genotype B[17]. Moreover, previous studies show that patients with genotype C harboured more rtA181T/V mutations than those with genotype B after antiviral treatment[24], and rtA181T/V was reported to reduce HBV susceptibility to LMV[39]. Therefore, Malmström et al.[38] were unable to determine whether the higher HBV loadings were due to the intrinsic properties of genotype C or the responsiveness of genotype C to antiviral drugs. Kuhnhenn et al.[40] failed to find the difference in HBV loadings among patients with CHB with genotypes A, B, C, D and E. Therefore, this claim that genotype C HBV has a higher replication level in the clinical setting is controversial. Masaya et al.[41] used the immunodeficient human-mouse chimeric liver model to prove that genotype C HBV with wild-type nt1896 in the pre-core region had a higher replication level, leading to more serious liver tissue damages than genotype-B HBV with wild-type nt1896. They further speculated that particular genetic variants of HBV could be directly cytopathic in immunosuppressive conditions. However, Masaya et al.[41] did not elucidate the mutation status of other nucleotide sites in isolated HBV strains other than nt1896, and thereby were unable to determine whether the virological and histological effects induced by genotype C HBV was a result of the intrinsic properties of genotype C or mutations harboured by isolated genotype C HBV strain. This study revealed that the mutation spectrum of the genotype C X gene clones was different from that of genotype B X gene clones, and the mutations in genotype C X gene clones were mainly clustered in the dimerization region and the aa110 to aa140 sites within the transcriptional activation region of HBx protein, which changes the function of HBx protein. Moreover, the mutation L123S located in the transactivation region was found to be significantly increased in tumour tissues, mainly existing in the genotype-C X gene clones.

Third, a novel mutant combination T81P/S101P/L123S was found, which conferred the tumour-promoting phenotype on HBx protein. HBx protein is encoded by the HBV X gene and can bind to transcription factors, playing a role in transactivation[42, 43]. Therefore, HBx is speculated to contribute to HCC development, but the mechanism underlying the tumour-promoting effect of HBx remains unclear. HBx has been reported to either mediate apoptosis to inhibit cell proliferation[43] or prevent apoptosis for cell survival[42]. Therefore, the effect of HBx protein on hepatocytes is still controversial. One explanation for this discrepancy is that mutations across the HBV X gene could affect the function of the HBx protein. Further, the transactivation activity of wild-type HBx could play a negative regulatory role on cell transformation; however, on the alteration of the aa residues of HBx protein, such as COOH-terminal truncation, HBx could exhibit a proliferation-promoting effect[21, 44]. Indeed, this study also showed that wild-type HBx could inhibit HCC cell proliferation compared with the control group in vivo and in vitro. Moreover, in a clinical setting, mutations across the HBV X gene have been reported to be closely related to HCC, and some mutations were reported to even increase the risk of HCC development in patients with CHB, which further highlights the importance of HBV X mutation in the development of HCC. Although quite a few mutations across the X gene have been reported in HBV-related patients with HCC, the phenotype-confirmed mutations are less in number, includingL129S/K130M/V131I[21], A10R/S144R[22] and F30V[23]. Among these, F30V and A10R/S144R were proved to attenuate the pro-apoptotic effect of HBx but not affect cell proliferation. However, there are many mutations across the X gene that are yet to be discovered and verified experimentally. In this study, mutation L123S was found to occur more frequently in tumour tissues compared with adjacent non-tumour tissues, mainly co-existing with S101P or T81P plus S101P in the same haplotype of the X gene. Further cell function experiments confirmed that HBx harbouring mutation T81P/T101P/L123S has a tumour-promoting function compared with wild-type HBx. Notably, the MYC protein, encoded by oncogene *c-myc,* was demonstrated to be a key mediator for the tumour-promoting effect of HBx-T81P/T101P/L123S.

However, this study does have certain limitations. First, owing to the difficulty in amplifying the full-length HBV genome in liver tissues, genotyping of the HBV X gene through phylogenetic analysis and an online genotyping tool was performed, which could introduce a slight difference in the results of genotyping based on the full-length genome but does not affect the overall conclusion. Second, the tumour-promoting effect of HBx-T81P/T101P/L123S was demonstrated using a subcutaneous xenograft model. Hence, further animal experiments based on the tail vein injection of a vector expressing HBx-T81P/T101P/L123S are required to directly verify the effect of HBx-T81P/T101P/L123S on inducing hepatocarcinogenesis, which could contribute to the construction of an HBV-related orthotopic liver cancer mouse model free of chemical and physical inducements. Finally, the clinical significance of mutation L123S needs further exploration using a large size of serum samples, which could facilitate the development of a potential viral marker for the monitoring of HCC.

## Supplementary Material

Supplemental Material
