# Supplementary figures and images for: Compartmentalisation of Hepatitis B virus X gene evolution in hepatocellular carcinoma microenvironment and the genotype-phenotype correlation of tumorigenicity in HBV-related patients with hepatocellular carcinoma
Source: Emerg Microbes Infect. 2022 Oct 26;11(1):2486–501. doi: 10.1080/22221751.2022.2125344 (PMC9621239; doi:10.1080/22221751.2022.2125344)

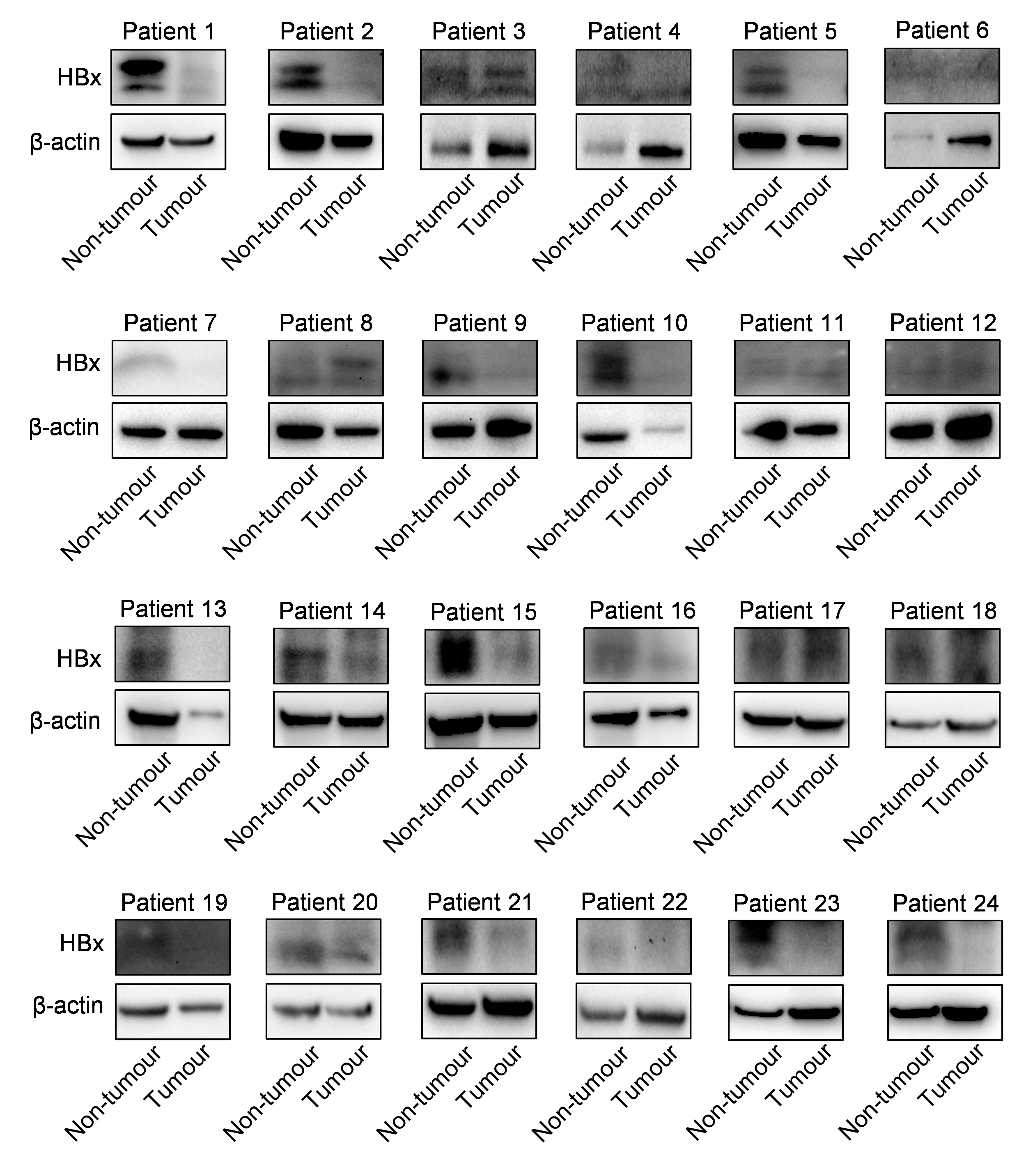

Supplement: Supplemental Material [file TEMI_A_2125344_SM2633.zip › Figure S1 _related to Table S1.tif]

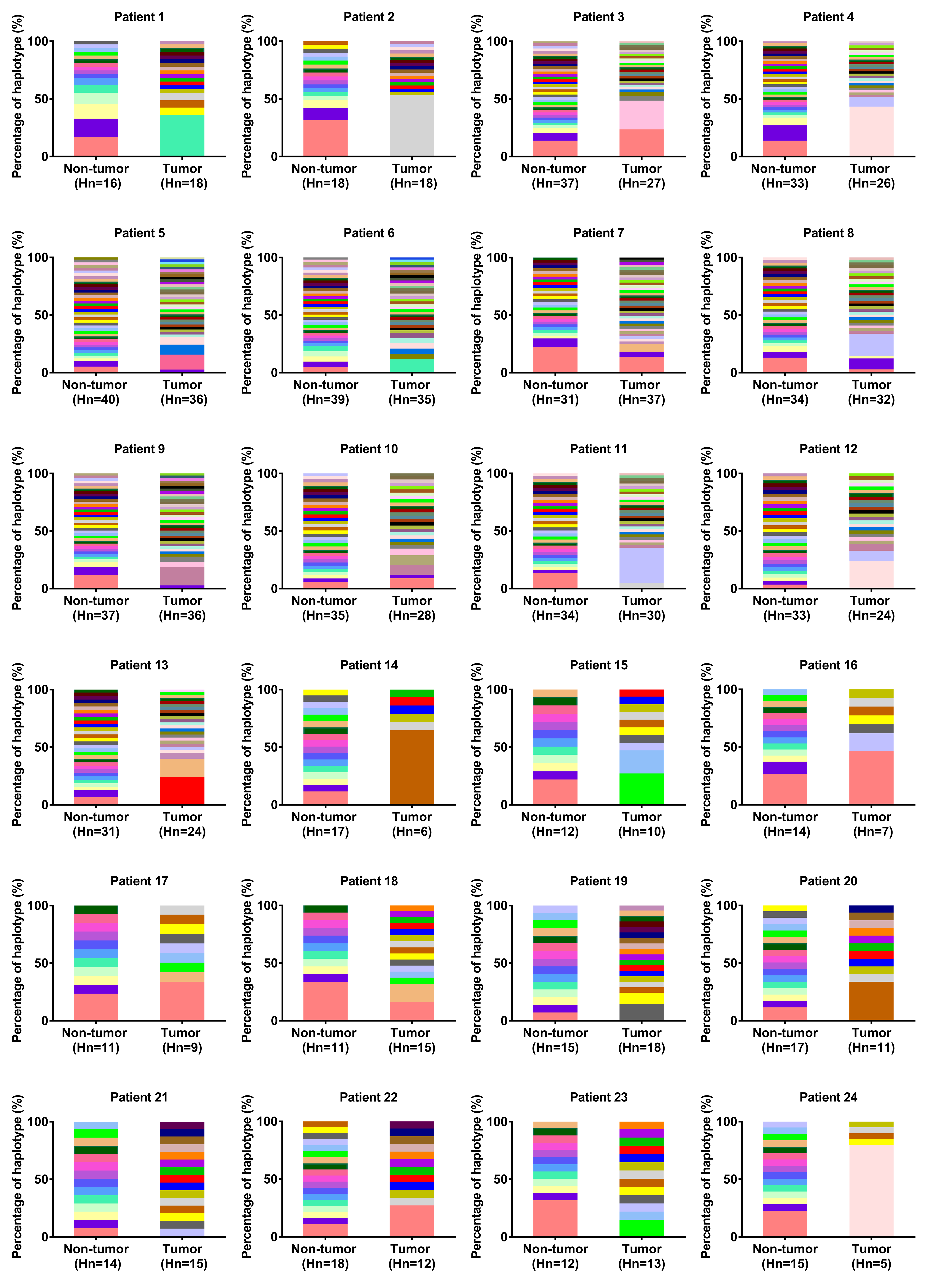

Supplement: Supplemental Material [file TEMI_A_2125344_SM2633.zip › Figure S2 _related to Figure 1A.tif]

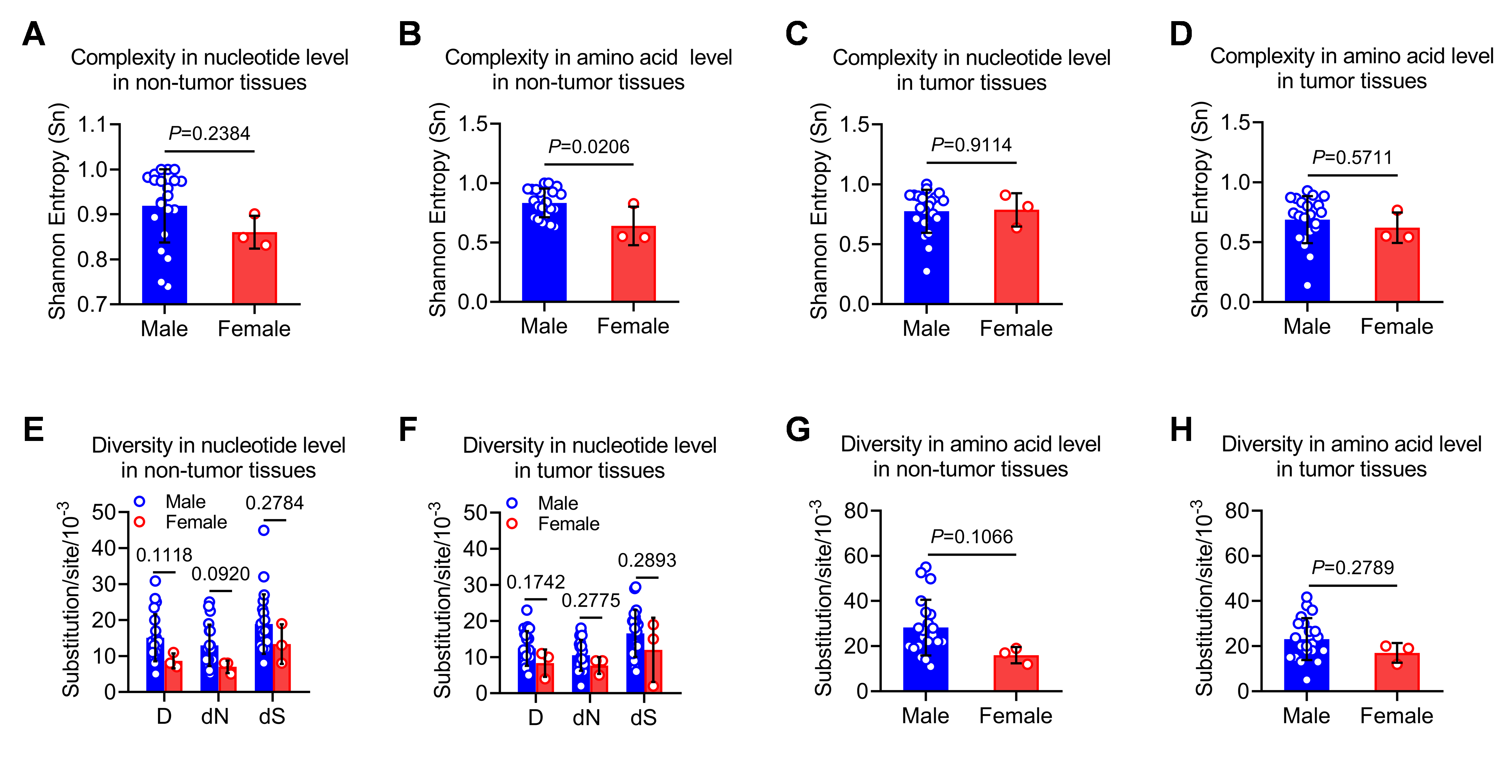

Supplement: Supplemental Material [file TEMI_A_2125344_SM2633.zip › Figure S3 _related to Figure 1B to 1E.tif]

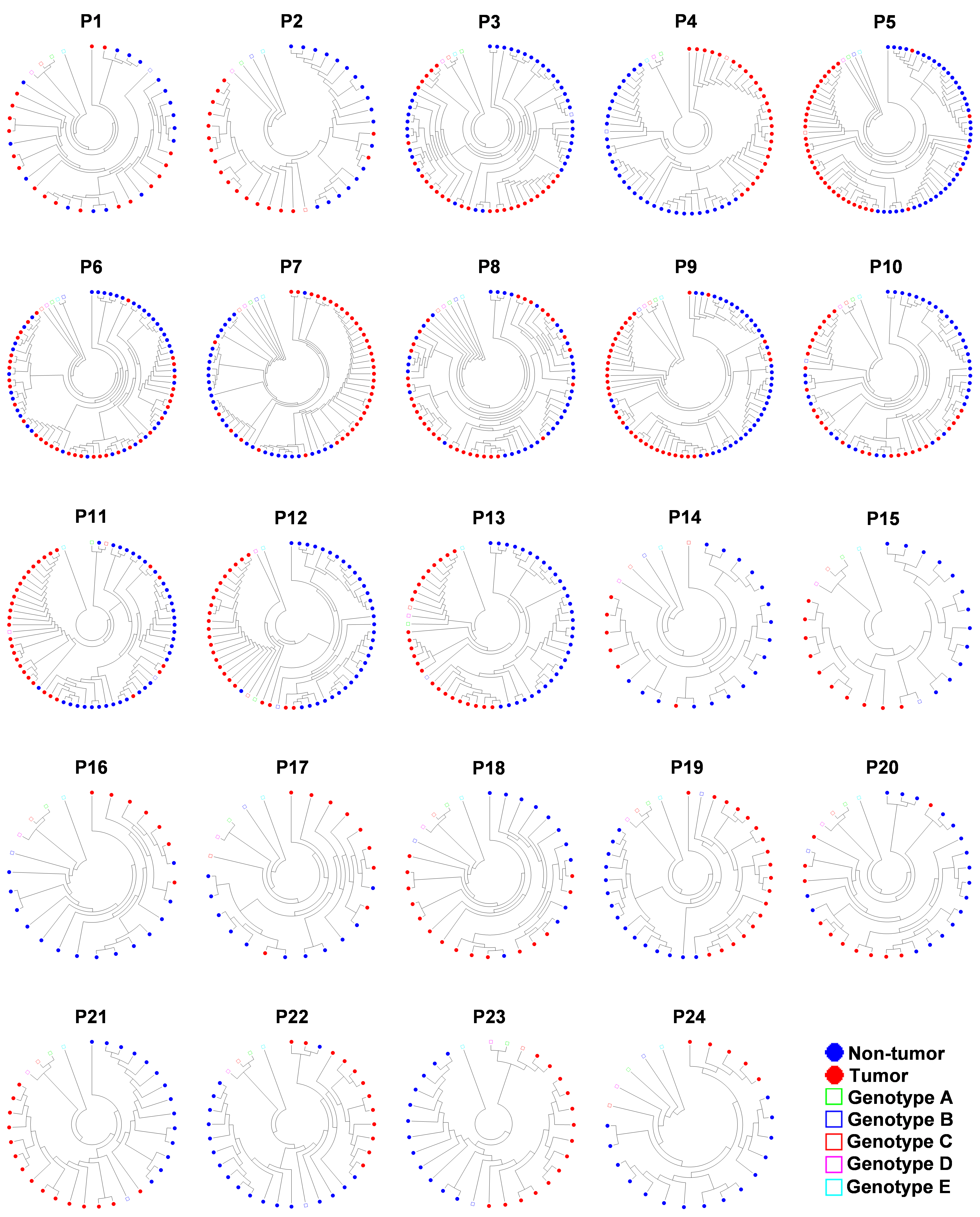

Supplement: Supplemental Material [file TEMI_A_2125344_SM2633.zip › Figure S4 _related to Figure 1F.tif]

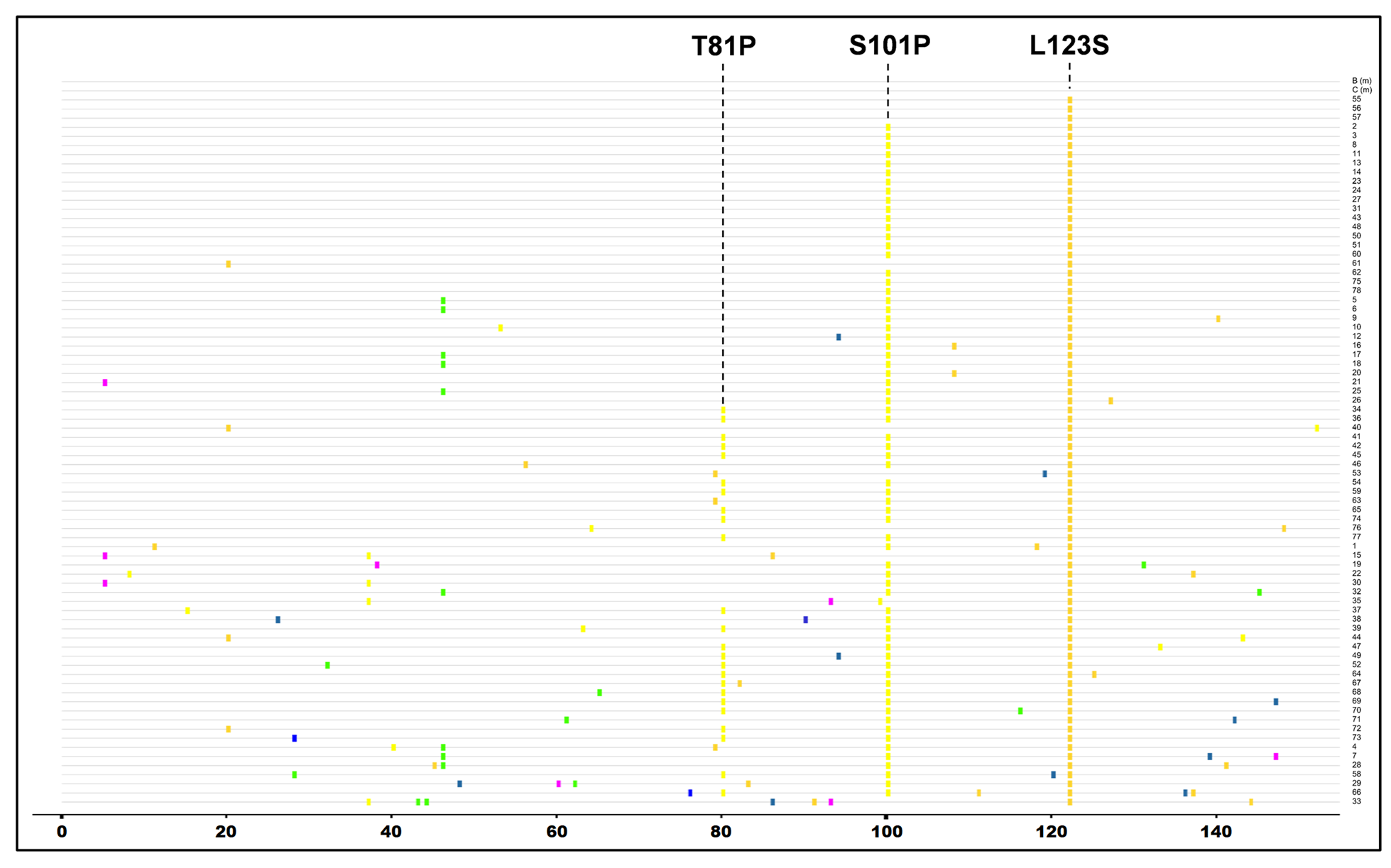

Supplement: Supplemental Material [file TEMI_A_2125344_SM2633.zip › Figure S5 _related to Figure 2L.tif]

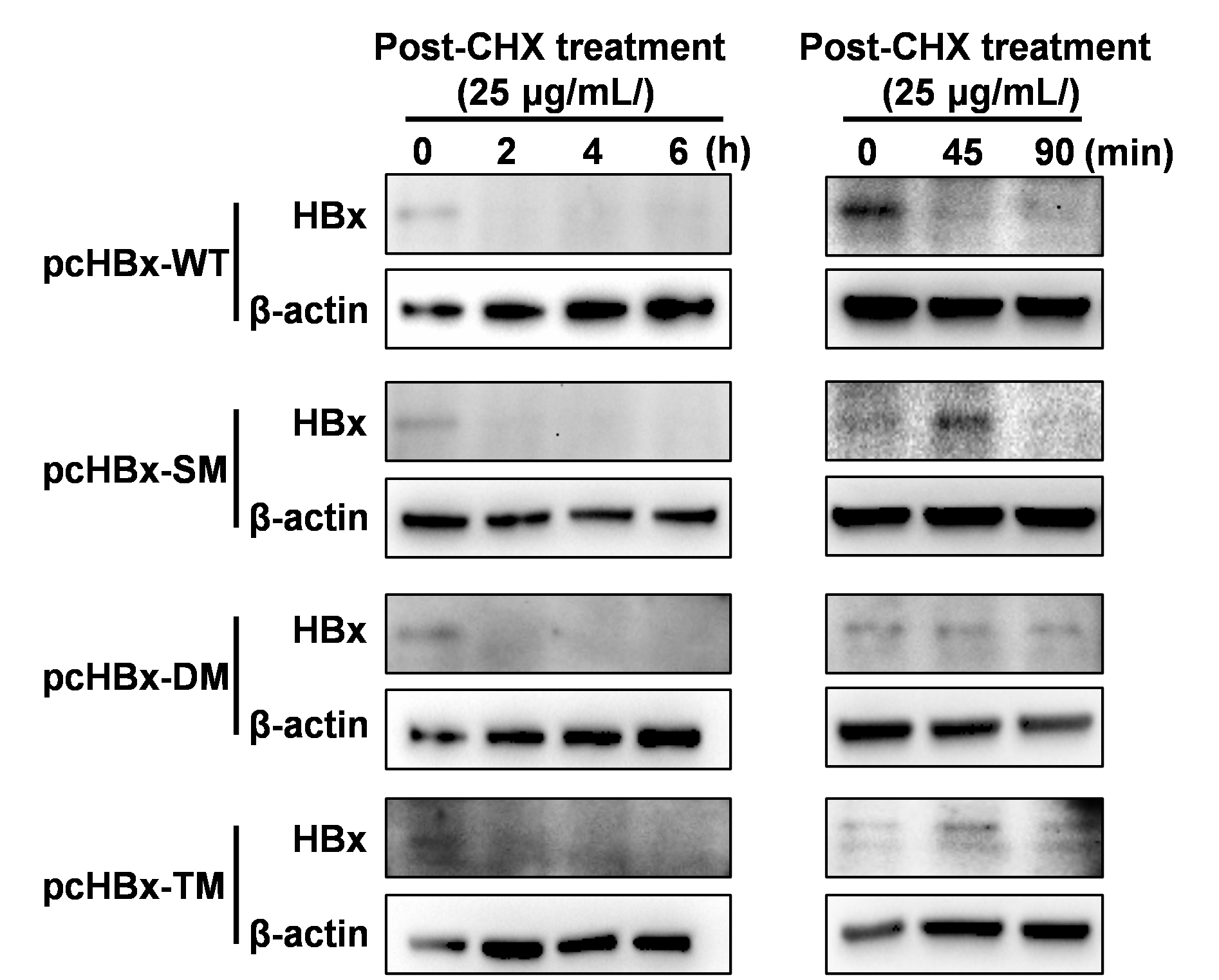

Supplement: Supplemental Material [file TEMI_A_2125344_SM2633.zip › Figure S6 _related to Figure 2N.tif]

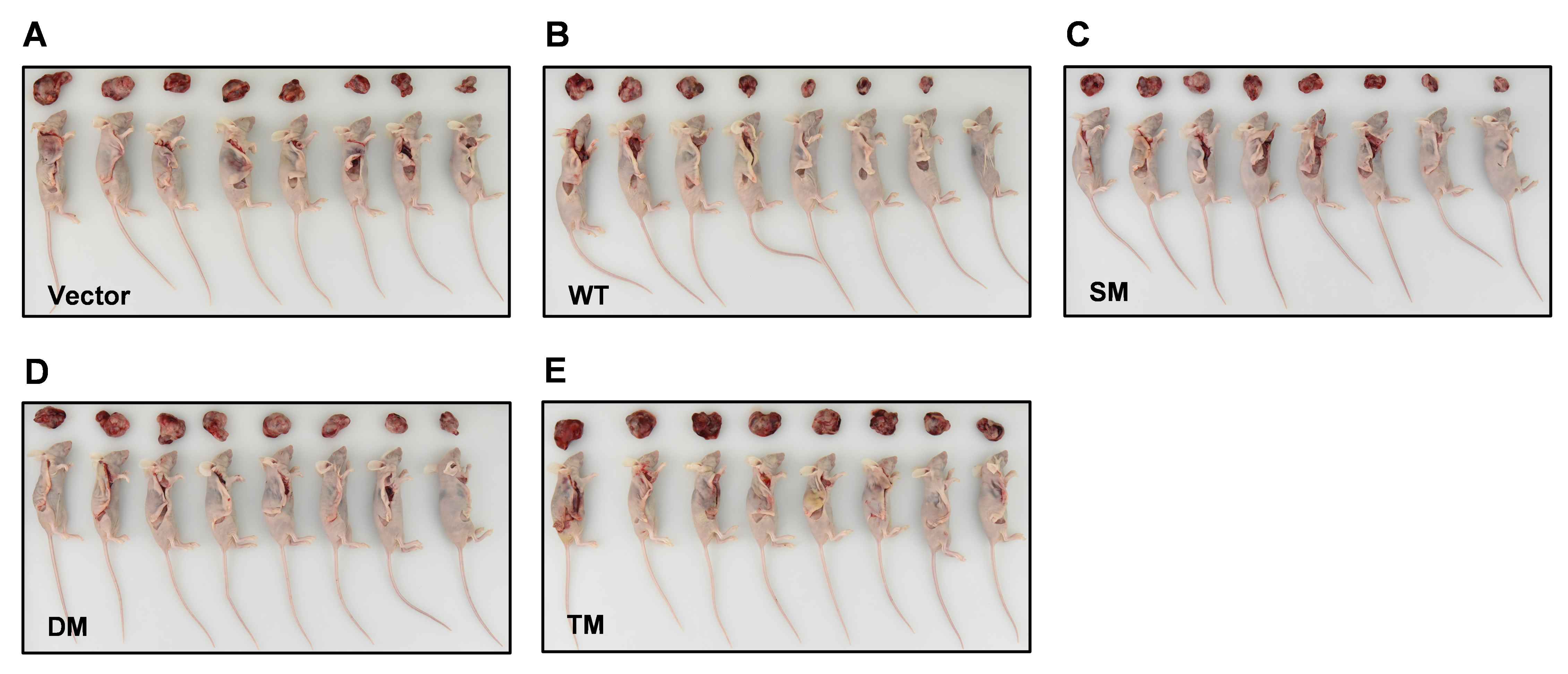

Supplement: Supplemental Material [file TEMI_A_2125344_SM2633.zip › Figure S7 _related to Figure 4A.tif]

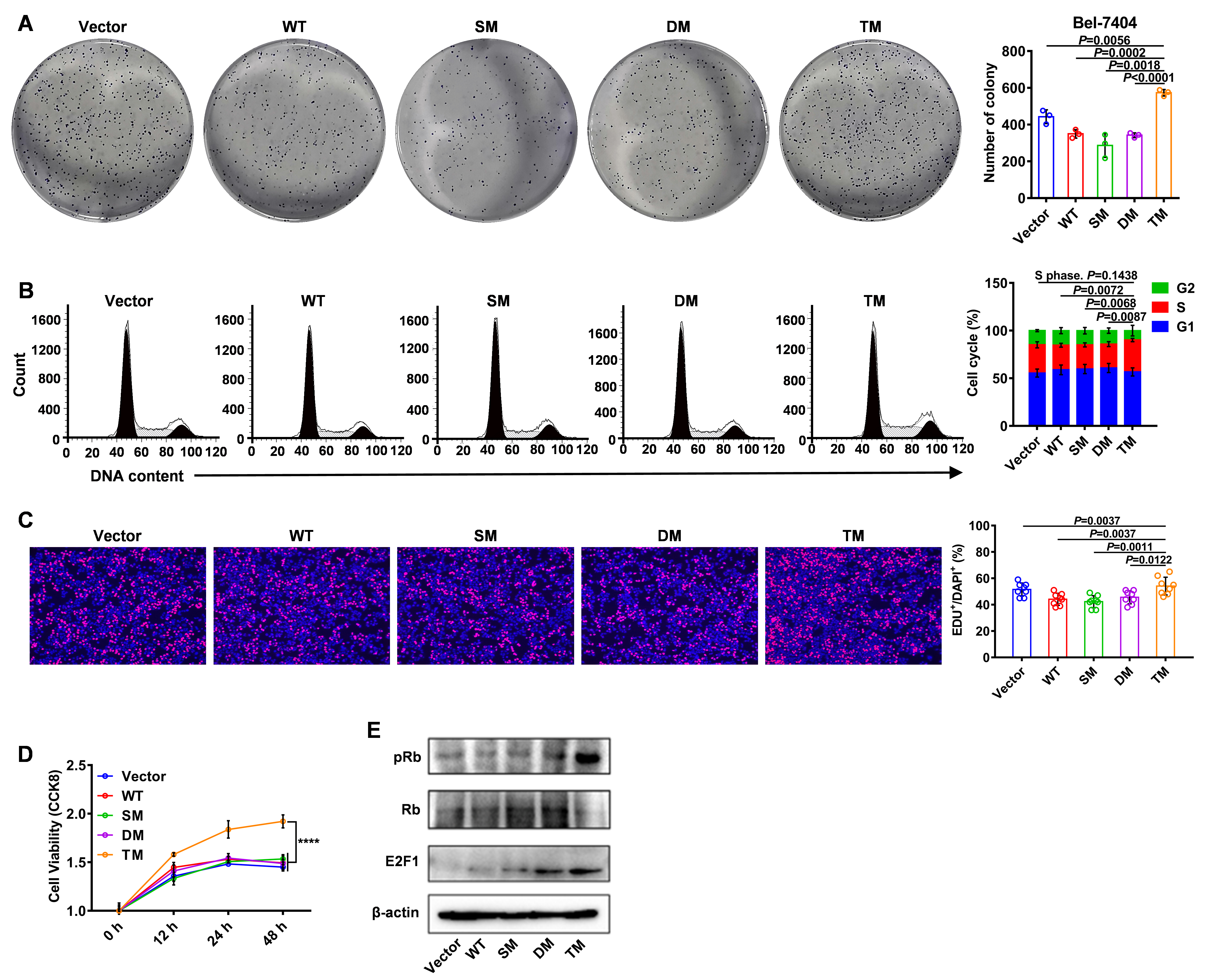

Supplement: Supplemental Material [file TEMI_A_2125344_SM2633.zip › Figure S8 _related to Figure 4D to 4H.tif]

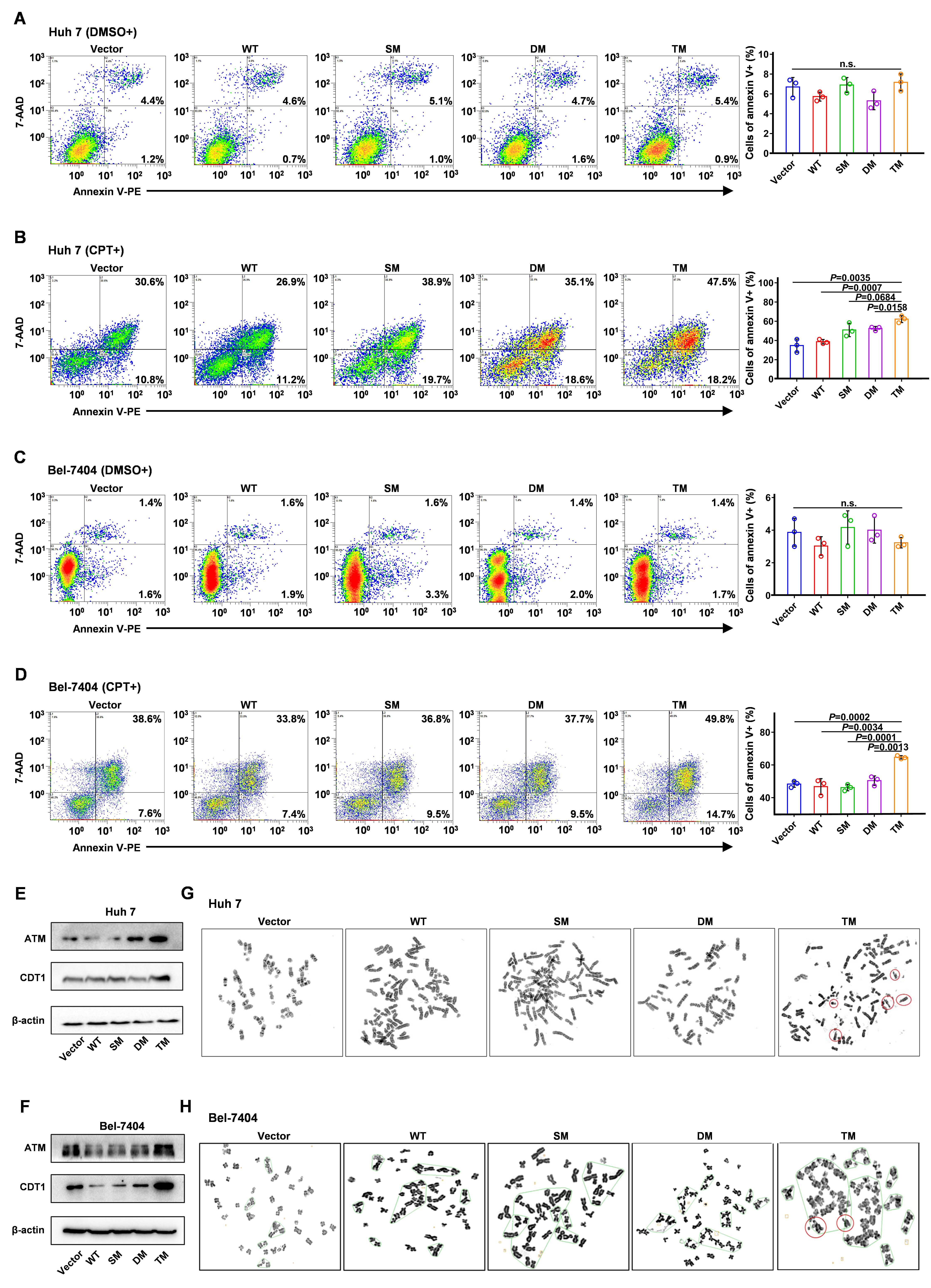

Supplement: Supplemental Material [file TEMI_A_2125344_SM2633.zip › Figure S9 _related to Figure 4.tif]
