# Supplementary material for: Compartmentalisation of Hepatitis B virus X gene evolution in hepatocellular carcinoma microenvironment and the genotype-phenotype correlation of tumorigenicity in HBV-related patients with hepatocellular carcinoma
Source: Emerg Microbes Infect. 2022 Oct 26;11(1):2486–501. doi: 10.1080/22221751.2022.2125344 (PMC9621239; doi:10.1080/22221751.2022.2125344)
Supplement: Supplemental Material [file TEMI_A_2125344_SM2633.zip › Supplemental Figure legends.doc]

Supplemental Figure legends

Supplementary Figure 1. Levels of HBV X proteins in the liver tissues in patients with HCC (related to Table S1)

Supplementary Figure 2. Patterns of haplotypes across the **Hepatitis B virus** (HBV) X gene in tumour and non-tumour tissues in each patient with hepatocellular carcinoma **(related to Figure 1)**

Representative patterns of haplotypes across HBV X gene in tumour and non-tumour tissues. The T-A cloning was used to isolate the HBV X gene. The Sanger sequencing was performed on each clone, and MEGA 7.0 software was used for the analysis of the haplotypes of the HBV X gene. Each intra-patient colour represents a type of haplotype. Hn, Haplotypes.

Supplementary Figure 3. **Comparisons of the heterogeneity of HBx haplotypes in liver tissues between male and female patients with HBV-related hepatocellular carcinoma (related to Figure 1B-1E)**

**(A-B)** The comparisons of the complexity quantified using Sn in nucleotide (A) and amino acid (B) level in non-tumour tissues. **(C-D)** The comparisons of the complexity quantified using Sn in nucleotide (C) and amino acid (D) level in tumour tissues. **(E-F)** The comparisons of the diversity quantified using D, dN and dS in nucleotide level in non-tumour (E) and tumour (F) tissues. **(G-H)** The comparisons of the complexity quantified using D in amino acid level in non-tumour (G) and tumour (H) tissues. Sn, Shannon entropy. D, Genetic distance. dS, Number of synonymous substitutions per synonymous site. dN, number of non-synonymous substitutions per non-synonymous site. Each symbols represent one patient; t test; n = 21 for male patients and n=3 for female patients.

Supplementary Figure 4. Phylogenetic trees of **Hepatitis B virus** (HBV) **quasispecies** across the X gene from liver tissues in each patient with hepatocellular carcinoma **(related to Figure 1F)**

Supplementary Figure 5. **Clones of HBV X gene with mutation L123S in tumour tissues (related to Figure 2M)**

Supplementary Figure 6. **Analysis of the half-life of wild- and mutant-type HBV X proteins in vitro (related to Figure 2N)**

Supplementary Figure 7. Tumour masses harvested from the nude mice injected subcutaneously with Huh 7 cells in each group **(related to Figure 4A)**

Supplementary Figure 8. **HBx-T81P/S101P/L123S promotes the growth and proliferation of** hepatocellular carcinoma **(HCC) cells compared with wild-type HBx in the Bel-7404 cell line (related to Figure 4D-4H)**

**(A)** Colony formation assays were performed to evaluate the single-cell growth ability in Bel-7404 cells. Differences in colony numbers among the Vector, WT, SM, DM and TM groups are presented on the right of the panel; Data are presented as mean ± standard error of the mean (SEM); Unpaired t test; n=3. **(B)** Cell cycles were analysed using flow cytometry in Bel-7404 cells. Proportions of cells in the S phase in the Vector, WT, SM, DM and TM groups are presented at the right of the panel; Each symbol represents an individual experiment. Data are presented as mean ± SEM; Unpaired t test; n=3. **(C)** Levels of DNA replication were examined using EdU incorporation assays in Bel-7404 cells. The proportions of cells with EdU staining in the Vector, WT, SM, DM and TM groups are presented at the right of the panel; Each symbol represents an individual experiment; Data are presented as mean ± SEM; Unpaired t test. n=3. **(D)** Cell viabilities of Bel-7404 cells were assessed using Cell counting kit-8 assays. Data are presented as mean ± SEM. Unpaired t test; n=6. **(F)** Protein levels of E2F1, Rb and pRb in Bel-7404 cells were assessed using Western blot.

Supplementary Figure 9**. HBx-T81P/S101P/L123S can increase the genomic instability of** hepatocellular carcinoma **(HCC) cells compared with wild-type HBx (related to Figure 4)**

**(A-D)** The dead cells were evaluated using flow cytometry in HCC cells. The camptothecin (CPT) genotoxicity was used to induce cell death in Huh 7 cells (B) and Bel-7404 cells (D). Dimethyl sulfoxide (DMSO) was used as a control in Huh 7 cells (A) and Bel-7404 cells (C). Annexin V-PE and 7-AAD were used to label the dead cells. The cells labelled with Annexin V-PE alone represent the viable apoptotic cells. The cells labelled with both Annexin V-PE and 7-AAD represent the non-viable apoptotic cells. The proportion of annexin V-PE (+) cells in Vector, WT, SM, DM and TM groups are presented on the right of the panel in (A-D) respectively. Each symbol represents an individual experiment. Data are presented as mean ± standard error of mean (SEM). Unpaired t test. n=3. **(E and F)** The protein levels of ATM and CDT1 were assessed using Western blot in Huh 7 cells (E) and Bel-7404 cells (F). β-actin was used as a loading control. ATM, Ataxia Telangiectasia Mutated. CDT1, Chromatin Licensing And DNA Replication Factor 1. **(G and H)** The representative chromosome karyotype analysis of cells in the metaphase in Huh 7 cells (G) and Bel-7404 cells (H). The chromosomes with breakages are indicated with red circles.
