# Supplementary material for: Compartmentalisation of Hepatitis B virus X gene evolution in hepatocellular carcinoma microenvironment and the genotype-phenotype correlation of tumorigenicity in HBV-related patients with hepatocellular carcinoma
Source: Emerg Microbes Infect. 2022 Oct 26;11(1):2486–501. doi: 10.1080/22221751.2022.2125344 (PMC9621239; doi:10.1080/22221751.2022.2125344)
Supplement: Supplemental Material [file TEMI_A_2125344_SM2633.zip › Table S1.doc]

| **Number** | **Gender** | **Age (year)** | **HBV DNA (IU/mL)** | **ALT (U/mL)** | **AST (U/mL)** | **HBsAg (IU/mL)** | **HBsAb (S/CO)** | **HBeAg (S/CO)** | **HBeAb (S/CO)** | **HBcAb (S/CO)** | **Anti-viral drug** | **HCV co-infection** |
| --- | --- | --- | --- | --- | --- | --- | --- | --- | --- | --- | --- | --- |
| **1** | male | 49 | 2.04E+06 | 53 | 38 | >250 | 0.05 | 1.8 | 1.27 | 10.13 | Entecavir | No |
| **2** | male | 57 | 2.15E+03 | 19 | 79 | 250 | 0.24 | 0.25 | 0.01 | 11.17 | - | No |
| **3** | male | 72 | 2.54E+05 | 151 | 110 | 37.55 | 12.44 | 0.25 | 0.01 | 7.58 | - | No |
| **4** | male | 51 | 3.87E+04 | 37 | 25 | >250 | 0 | 0.33 | 0.01 | 5.59 | Entecavir | No |
| **5** | male | 60 | 6.02E+03 | 462 | 384 | 517.8 | 4.08 | 0.35 | 0.55 | 5.53 | Entecavir | No |
| **6** | male | 53 | 9.78E+02 | 60 | 74 | 130.26 | 0.02 | 0.63 | 1.45 | 6.59 | Entecavir | No |
| **7** | female | 57 | 5.44E+04 | 40 | 45 | >250 | 0 | 0.32 | 0.01 | 7.55 | Entecavir | No |
| **8** | male | 41 | 2.66E+03 | 44 | 64 | >250 | 2.38 | 2.08 | 0.52 | 10.54 | Entecavir | No |
| **9** | male | 67 | 1.16E+06 | 22 | 43 | >250 | 0 | 0.33 | 0.01 | 10.32 | Entecavir | No |
| **10** | male | 59 | 2.45E+06 | 38 | 95 | >250 | 0 | 544.2 | 27.79 | 9.6 | - | No |
| **11** | male | 58 | 6.94E+02 | 15 | 24 | 242.14 | 0.43 | 0.34 | 0.01 | 11.85 | Entecavir | No |
| **12** | male | 53 | 7.83E+02 | 19 | 42 | >250 | 0 | 0.33 | 0.01 | 10.35 | - | No |
| **13** | male | 56 | 5.88E+02 | 52 | 37 | >250 | 0 | 0.26 | 0.01 | 17.6 | - | No |
| **14** | male | 60 | 4.88E+03 | 24 | 23 | 165.78 | 0 | 0.31 | 0.01 | 10.89 | Entecavir | No |
| **15** | male | 48 | 1.20E+07 | 45 | 48 | >250 | 0.84 | 256.67 | 10.29 | 8.6 | Entecavir | No |
| **16** | female | 57 | 5.96E+02 | 17 | 31 | 88.68 | 0 | 0.67 | 1.63 | 9.8 | - | No |
| **17** | female | 72 | 1.06E+05 | 65 | 40 | >250 | 0.77 | 0.32 | 0.01 | 12.66 | Entecavir | No |
| **18** | male | 49 | 2.59E+05 | 154 | 65 | >250 | 0.19 | 0.56 | 0.53 | 17.9 | Entecavir | No |
| **19** | male | 32 | 8.10E+02 | 43 | 39 | >250 | 0.89 | 0.38 | 0.01 | 11.61 | Entecavir | No |
| **20** | male | 48 | 4.00E+04 | 193 | 99 | >250 | 0.23 | 6.26 | 1.3 | 8.75 | Entecavir | No |
| **21** | male | 59 | 2.03E+06 | 42 | 74 | >250 | 0.85 | 0.37 | 0.01 | 12.76 | Entecavir | No |
| **22** | male | 38 | 1.21E+05 | 41 | 83 | >250 | 0.22 | 0.27 | 0.01 | 13.31 | Entecavir | No |
| **23** | male | 51 | 1.32E+05 | 597 | 163 | >250 | 0.09 | 0.33 | 0.01 | 12.42 | - | No |
| **24** | male | 45 | 1.02E+07 | 59 | 39 | >250 | 0 | 32.16 | 2.43 | 13.12 | Entecavir | No |

**Table S1. Clinical characteristics of HCC patients in this study**
