# Supplementary material for: Compartmentalisation of Hepatitis B virus X gene evolution in hepatocellular carcinoma microenvironment and the genotype-phenotype correlation of tumorigenicity in HBV-related patients with hepatocellular carcinoma
Source: Emerg Microbes Infect. 2022 Oct 26;11(1):2486–501. doi: 10.1080/22221751.2022.2125344 (PMC9621239; doi:10.1080/22221751.2022.2125344)
Supplement: Supplemental Material [file TEMI_A_2125344_SM2633.zip › Table S2.doc]

**Table S2. Mutation characteristics of HBV X clones in tumor and non-tumor tissues**

| **Position** | **Non-tumor tissues (n=680)** | | **Tumor tissues (n=711)** | |
| --- | --- | --- | --- | --- |
|  | **Most common (may be multiple)** | **Others** | **Most common (may be multiple)** | **Others** |
| 1 | M(664) | I(6) L(4) T(4) V(2) | M(707) | I(1) T(1) V(2) |
| 2 | A(670) | T(8) V(2) | A(711) | - |
| 3 | A(677) | T(3) | A(705) | D(1) T(5) |
| 4 | R(671) | G(6) K(3) | R(660) | G(44) W(7) |
| 5 | L(423) | A(1) M(78) P(2) R(1) T(1) V(174) | L(395) | A(1) M(73) V(242) |
| 6 | C(620) | R(2) S(1) Y(57) | C(680) | R(2) Y(29) |
| 7 | C(675) | R(2) Y(3) | C(704) | R(5) Y(2) |
| 8 | Q(676) | R(4) | Q(697) | H(2) P(6) R(6) |
| 9 | L(679) | P(1) | L(704) | P(7) |
| 10 | D(667) | A(1) E(1) G(1) H(1) N(8) Y(1) | D(704) | E(1) G(4) N(2) |
| 11 | P(680) | - | P(710) | S(1) |
| 12 | A(569) | T(110) V(1) | A(613) | T(97) V(1) |
| 13 | R(679) | W(1) | R(709) | G(1) Q(1) |
| 14 | D(672) | G(4) N(4) | D(708) | G(2) N(1) |
| 15 | V(675) | A(2) D(1) I(2) | V(705) | A(2) G(1) I(3) |
| 16 | L(674) | F(1) P(5) | L(709) | H(1) P(1) |
| 17 | C(662) | R(15) S(1) W(1) Y(1) | C(706) | R(2) W(1) Y(2) |
| 18 | L(675) | F(2) S(1) V(2) | L(708) | F(3) |
| 19 | R(680) | - | R(711) | - |
| 20 | P(680) | - | P(708) | L(2) S(1) |
| 21 | V(664) | A(3) L(13) | V(689) | A(9) I(1) L(12) |
| 22 | G(673) | E(5) S(2) | G(709) | D(1) S(1) |
| 23 | A(674) | P(1) T(4) V(1) | A(710) | V(1) |
| 24 | E(674) | D(5) K(1) | E(708) | G(1) K(2) |
| 25 | S(679) | P(1) | S(706) | P(5) |
| 26 | R(678) | H(1) P(1) | R(676) | C(34) H(1) |
| 27 | G(672) | deletion(5) E(1) R(2) | G(710) | R(1) |
| 28 | R(674) | deletion(5) P(1) | R(709) | *(2) |
| 29 | P(632) | deletion(5) S(43) | P(650) | H(1) L(1) S(59) |
| 30 | L(425) | deletion(7) A(1) P(1) V(246) | L(357) | F(34) H(1) V(319) |
| 31 | P(363) | deletion(5) A(26) L(1) S(234) T(51) | S(339) | A(3) L(1) P(312) T(56) |
| 32 | G(598) | deletion(6) R(76) | G(635) | R(76) |
| 33 | P(668) | deletion(5) Q(1) S(2) T(4) | P(675) | L(1) S(34) T(1) |
| 34 | L(428) | deletion(5) F(233) P(1) V(13) | L(391) | F(306) P(1) S(1) V(12) |
| 35 | G(671) | deletion(5) E(1) R(2) W(1) | G(702) | R(9) |
| 36 | A(507) | deletion(5) G(1) H(1) N(1) P(64) S(15) T(86) | A(525) | P(62) S(12) T(112) |
| 37 | L(673) | deletion(5) I(1) Q(1) | L(707) | I(1) P(2) Q(1) |
| 38 | P(554) | deletion(5) L(1) S(120) | P(538) | L(1) Q(1) S(171) |
| 39 | P(431) | deletion(5) A(1) S(243) | P(359) | F(1) S(351) |
| 40 | A(416) | deletion(5) H(1) P(235) S(14) T(8) V(1) | A(357) | P(341) S(12) T(1) |
| 41 | S(673) | deletion(5) P(2) | S(709) | P(2) |
| 42 | P(426) | deletion(5) A(70) S(178) T(1) | P(394) | A(63) S(254) |
| 43 | P(414) | deletion(5) S(261) | P(358) | S(353) |
| 44 | V(328) | deletion(5) A(262) G(1) I(14) L(56) P(1) R(13) | A(354) | deletion(1) F(24) I(12) L(106) R(34) V(180) |
| 45 | V(603) | deletion(6) A(1) I(14) L(55) Q(1) | V(664) | deletion(1) A(1) L(45) |
| 46 | P(670) | deletion(5) L(1) Q(1) S(2) T(1) | P(707) | deletion(1) L(2) S(1) |
| 47 | T(422) | deletion(5) A(252) I(1) | T(386) | deletion(1) A(310) P(2) S(3) V(9) |
| 48 | D(652) | deletion(5) A(5) N(16) V(2) | D(698) | deletion(2) E(1) G(4) N(6) |
| 49 | H(633) | deletion(6) R(4) Y(37) | H(664) | deletion(1) R(3) Y(43) |
| 50 | G(663) | deletion(6) E(1) R(10) | G(707) | deletion(1) E(1) R(2) |
| 51 | A(673) | deletion(6) V(1) | A(708) | deletion(2) V(1) |
| 52 | H(670) | deletion(1) N(1) R(6) Y(2) | H(684) | deletion(2) N(14) R(4) Y(7) |
| 53 | L(674) | deletion(1) F(1) H(2) P(2) | L(706) | deletion(2) F(1) H(1) P(1) |
| 54 | S(675) | deletion(1) P(4) | S(707) | deletion(2) P(2) |
| 55 | L(674) | deletion(2) F(1) I(1) S(1) V(1) | L(706) | deletion(3) F(1) V(1) |
| 56 | R(678) | deletion(1) H(1) | R(706) | deletion(2) C(1) H(2) |
| 57 | G(669) | deletion(1) E(3) R(7) | G(706) | deletion(2) D(1) R(1) S(1) |
| 58 | L(676) | deletion(1) H(2) P(1) | L(708) | deletion(2) P(1) |
| 59 | P(677) | deletion(1) L(2) | P(702) | deletion(2) L(7) |
| 60 | V(641) | deletion(1) A(1) I(37) | V(682) | deletion(2) A(2) F(1) I(24) |
| 61 | C(671) | deletion(1) F(7) R(1) | C(707) | deletion(2) F(1) Y(1) |
| 62 | A(679) | deletion(1) | A(707) | deletion(1) T(1) V(2) |
| 63 | F(672) | deletion(1) L(2) S(5) | F(704) | deletion(1) L(5) S(1) |
| 64 | S(675) | deletion(1) P(4) | S(703) | deletion(1) L(3) P(4) |
| 65 | S(674) | deletion(1) F(1) P(4) | S(703) | deletion(4) P(4) |
| 66 | A(659) | deletion(1) I(1) T(19) | A(699) | deletion(4) T(7) V(1) |
| 67 | G(669) | *(7) E(2) R(1) T(1) | G(702) | *(1) deletion(4) R(4) |
| 68 | P(677) | L(1) S(2) | P(707) | deletion(4) |
| 69 | C(676) | deletion(1) R(1) S(1) Y(1) | C(703) | deletion(4) R(3) Y(1) |
| 70 | A(666) | P(1) S(1) T(10) V(2) | A(705) | deletion(4) T(1) V(1) |
| 71 | L(675) | F(1) P(4) | L(706) | deletion(4) P(1) |
| 72 | R(669) | deletion(8) H(2) P(1) | R(705) | deletion(5) H(1) |
| 73 | F(657) | deletion(8) L(11) S(3) V(1) | F(694) | deletion(5) I(1) L(8) S(2) V(1) |
| 74 | T(666) | deletion(8) A(5) I(1) | T(702) | deletion(5) A(4) |
| 75 | S(669) | deletion(8) A(1) P(2) | S(702) | deletion(5) F(1) P(3) |
| 76 | A(677) | T(3) | A(706) | deletion(4) V(1) |
| 77 | R(679) | deletion(1) | R(706) | deletion(3) C(1) H(1) |
| 78 | R(676) | deletion(1) C(1) H(2) | R(705) | deletion(3) C(3) |
| 79 | M(673) | deletion(1) K(1) T(2) V(3) | M(705) | deletion(3) T(1) V(2) |
| 80 | E(653) | A(18) D(1) G(5) V(3) | E(687) | A(19) G(3) K(1) V(1) |
| 81 | T(673) | A(5) I(2) | T(686) | A(1) P(24) |
| 82 | T(674) | A(6) | T(710) | A(1) |
| 83 | V(679) | M(1) | V(705) | A(5) M(1) |
| 84 | N(672) | D(3) S(4) Y(1) | N(703) | D(1) S(7) |
| 85 | A(648) | deletion(1) D(11) H(19) T(1) | A(673) | H(38) |
| 86 | H(668) | deletion(1) L(1) P(10) | H(708) | deletion(1) R(1) Y(1) |
| 87 | R(368) | G(52) Q(254) W(6) | R(348) | deletion(1) G(39) Q(323) |
| 88 | N(429) | deletion(1) A(21) D(1) H(1) V(227) | N(358) | A(2) D(3) M(26) S(2) V(319) Y(1) |
| 89 | L(675) | deletion(1) P(1) Q(1) S(2) | L(705) | P(5) S(1) |
| 90 | P(679) | deletion(1) | P(711) | - |
| 91 | K(674) | deletion(1) E(2) Q(1) R(2) | K(707) | E(2) R(2) |
| 92 | V(673) | deletion(1) A(6) | V(710) | A(1) |
| 93 | L(675) | deletion(2) S(2) W(1) | L(710) | S(1) |
| 94 | H(522) | deletion(1) C(1) R(3) S(1) Y(152) | H(516) | Y(195) |
| 95 | K(666) | deletion(2) E(1) Q(6) R(5) | K(706) | E(1) Q(1) R(3) |
| 96 | R(674) | deletion(1) G(1) K(1) W(3) | R(706) | G(5) |
| 97 | T(663) | A(2) I(2) S(13) | T(696) | A(3) S(12) |
| 98 | L(675) | F(2) I(1) P(1) R(1) | L(708) | H(1) I(1) P(1) |
| 99 | G(674) | E(1) R(4) V(1) | G(694) | E(16) R(1) |
| 100 | L(677) | P(3) | L(708) | P(3) |
| 101 | S(602) | deletion(1) P(77) | S(625) | P(86) |
| 102 | A(661) | T(6) V(13) | A(709) | T(2) |
| 103 | M(660) | K(1) L(17) T(1) V(1) | M(710) | T(1) |
| 104 | S(678) | L(1) P(1) | S(708) | P(3) |
| 105 | T(667) | A(1) M(12) | T(707) | A(3) M(1) |
| 106 | T(674) | A(6) | T(709) | A(2) |
| 107 | D(674) | G(5) N(1) | D(711) | - |
| 108 | L(675) | F(1) I(1) P(3) | L(707) | F(1) P(3) |
| 109 | E(674) | G(5) K(1) | E(705) | G(6) |
| 110 | A(673) | E(1) T(3) V(3) | A(710) | T(1) |
| 111 | Y(675) | deletion(1) H(4) | Y(695) | C(14) H(1) N(1) |
| 112 | F(673) | L(3) S(4) | F(707) | L(2) S(2) |
| 113 | K(677) | E(1) N(1) R(1) | K(708) | E(3) |
| 114 | D(672) | G(4) N(4) | D(708) | E(1) G(1) N(1) |
| 115 | C(675) | *(1) R(3) Y(1) | C(706) | R(1) S(1) Y(3) |
| 116 | V(575) | A(3) L(99) M(2) S(1) | V(587) | L(123) S(1) |
| 117 | F(676) | L(2) S(2) | F(709) | L(1) S(1) |
| 118 | K(313) | A(3) N(66) R(1) T(297) | K(407) | I(2) N(9) T(293) |
| 119 | E(428) | D(250) G(2) | E(361) | A(1) D(346) G(3) |
| 120 | W(673) | *(4) C(1) R(2) | W(708) | *(1) R(2) |
| 121 | E(673) | G(4) K(3) | E(706) | G(1) K(3) V(1) |
| 122 | E(674) | D(3) G(2) K(1) | E(708) | D(1) G(1) K(1) |
| 123 | L(631) | S(49) | L(634) | S(77) |
| 124 | G(670) | E(1) L(6) R(3) | G(711) |  |
| 125 | E(611) | deletion(35) A(7) G(6) I(6) K(2) L(13) | E(685) | deletion(23) G(2) K(1) |
| 126 | E(636) | deletion(23) G(2) I(13) V(6) | E(670) | deletion(35) D(3) G(3) |
| 127 | I(469) | deletion(25) D(1) F(28) L(8) M(26) N(13) S(4) T(46) V(60) | I(472) | deletion(36) A(1) F(16) M(26) N(2) T(51) V(107) |
| 128 | R(627) | deletion(39) G(6) K(2) N(6) | R(675) | deletion(35) G(1) |
| 129 | L(625) | deletion(45) G(6) I(3) S(1) | L(688) | deletion(22) S(1) |
| 130 | M(373) | deletion(46) I(7) K(239) N(7) T(1) V(7) | M(444) | deletion(41) I(18) K(205) N(2) T(1) |
| 131 | I(412) | deletion(45) F(1) T(2) V(220) | I(471) | deletion(41) A(4) M(1) T(1) V(193) |
| 132 | F(616) | *(1) deletion(31) C(3) L(1) S(3) V(6) Y(19) | F(652) | deletion(42) C(1) L(3) S(5) Y(8) |
| 133 | V(670) | deletion(2) A(1) I(7) | V(696) | deletion(14) G(1) |
| 134 | L(679) | P(1) | L(687) | deletion(2) I(20) P(1) R(1) |
| 135 | G(678) | E(2) | G(709) | deletion(2) |
| 136 | G(678) | D(2) | G(710) | D(1) |
| 137 | C(677) | R(3) | C(710) | R(1) |
| 138 | R(672) | G(3) K(4) W(1) | R(709) | G(2) |
| 139 | H(678) | R(1) Y(1) | H(709) | R(1) Y(1) |
| 140 | K(675) | N(1) R(2) T(2) | K(708) | E(1) R(1) T(1) |
| 141 | L(679) | S(1) | L(707) | deletion(1) S(3) |
| 142 | V(675) | A(4) M(1) | V(708) | A(3) |
| 143 | C(652) | R(27) S(1) | C(698) | R(12) Y(1) |
| 144 | S(675) | L(3) P(2) | S(680) | A(27) P(4) |
| 145 | P(646) | L(1) Q(1) S(32) | P(667) | S(44) |
| 146 | A(660) | deletion(1) S(17) T(2) | A(708) | S(1) T(1) V(1) |
| 147 | P(676) | deletion(1) T(3) | P(711) | - |
| 148 | C(664) | deletion(12) R(3) W(1) | C(701) | G(1) R(6) W(2) Y(1) |
| 149 | N(666) | deletion(11) C(1) I(1) S(1) | N(700) | D(1) I(1) S(8) T(1) |
| 150 | F(672) | deletion(2) I(1) L(3) S(1) V(1) | F(704) | deletion(2) I(1) L(3) V(1) |
| 151 | F(641) | deletion(9) L(27) S(2) Y(1) | F(680) | L(6) S(5) V(19) Y(1) |
| 152 | T(668) | deletion(3) A(1) I(1) P(4) S(3) | T(709) | I(1) P(1) |
| 153 | S(674) | deletion(4) F(1) P(1) | S(690) | P(21) |
| 154 | A(675) | deletion(1) P(1) V(3) | A(708) | T(1) V(2) |
| 155 | *(675) | L(1) Q(1) Y(3) | *(708) | Q(2) Y(1) |

*, stop codon
