# Supplementary material for: Compartmentalisation of Hepatitis B virus X gene evolution in hepatocellular carcinoma microenvironment and the genotype-phenotype correlation of tumorigenicity in HBV-related patients with hepatocellular carcinoma
Source: Emerg Microbes Infect. 2022 Oct 26;11(1):2486–501. doi: 10.1080/22221751.2022.2125344 (PMC9621239; doi:10.1080/22221751.2022.2125344)
Supplement: Supplemental Material [file TEMI_A_2125344_SM2633.zip › Table S3.doc]

| **Table S3. Entropy difference in HBV X between tumor and non-tumor tissues** | | | | | |
| --- | --- | --- | --- | --- | --- |
| **Position** | **Query consensus** | **Entropy difference** | **randomized entropy diff>=Hdiff** | **Highest randomized entropy diff** | **P-value** |
| 1 | M | 0.102 | 1 | 0.103 | 0.01 |
| 2 | A | 0.084 | 0 | 0.073 | 0.00 |
| 3 | A | -0.024 | 29 | 0.062 | 0.29 |
| 4 | R | -0.208 | 0 | 0.12 | 0.00 |
| 5 | L | 0.002 | 97 | 0.101 | 0.97 |
| 6 | C | 0.129 | 0 | 0.093 | 0.00 |
| 7 | C | -0.013 | 59 | 0.096 | 0.59 |
| 8 | Q | -0.081 | 2 | 0.085 | 0.02 |
| 9 | L | -0.044 | 6 | 0.064 | 0.06 |
| 10 | D | 0.054 | 12 | 0.089 | 0.12 |
| 11 | P | -0.011 | 42 | 0.02 | 0.42 |
| 12 | A | 0.045 | 24 | 0.089 | 0.24 |
| 13 | R | -0.01 | 62 | 0.051 | 0.62 |
| 14 | D | 0.042 | 11 | 0.06 | 0.11 |
| 15 | V | -0.006 | 88 | 0.068 | 0.88 |
| 16 | L | 0.033 | 14 | 0.074 | 0.14 |
| 17 | C | 0.09 | 0 | 0.086 | 0.00 |
| 18 | L | 0.024 | 35 | 0.071 | 0.35 |
| 19 | R | 0 | 100 | 0 | 1.00 |
| 20 | P | -0.03 | 12 | 0.047 | 0.12 |
| 21 | V | -0.041 | 27 | 0.092 | 0.27 |
| 22 | G | 0.042 | 10 | 0.069 | 0.10 |
| 23 | A | 0.048 | 7 | 0.071 | 0.07 |
| 24 | E | 0.025 | 44 | 0.076 | 0.44 |
| 25 | S | -0.031 | 11 | 0.068 | 0.11 |
| 26 | R | -0.18 | 0 | 0.082 | 0.00 |
| 27 | G | 0.064 | 3 | 0.073 | 0.03 |
| 28 | R | 0.054 | 2 | 0.062 | 0.02 |
| 29 | P | -0.028 | 48 | 0.102 | 0.48 |
| 30 | L | -0.126 | 0 | 0.092 | 0.00 |
| 31 | S | 0.12 | 0 | 0.096 | 0.00 |
| 32 | G | 0.064 | 12 | 0.096 | 0.12 |
| 33 | P | -0.103 | 1 | 0.113 | 0.01 |
| 34 | L | 0.001 | 97 | 0.073 | 0.97 |
| 35 | G | 0.018 | 54 | 0.083 | 0.54 |
| 36 | A | 0.055 | 29 | 0.154 | 0.29 |
| 37 | L | 0.025 | 42 | 0.072 | 0.42 |
| 38 | P | -0.053 | 17 | 0.086 | 0.17 |
| 39 | P | 0 | 99 | 0.076 | 0.99 |
| 40 | A | 0.079 | 2 | 0.093 | 0.02 |
| 41 | S | 0.044 | 9 | 0.064 | 0.09 |
| 42 | P | 0.014 | 67 | 0.105 | 0.67 |
| 43 | P | 0.013 | 46 | 0.043 | 0.46 |
| 44 | A | -0.181 | 0 | 0.124 | 0.00 |
| 45 | V | 0.198 | 0 | 0.155 | 0.00 |
| 46 | P | 0.056 | 8 | 0.079 | 0.08 |
| 47 | T | -0.088 | 1 | 0.088 | 0.01 |
| 48 | D | 0.103 | 3 | 0.126 | 0.03 |
| 49 | H | 0.031 | 49 | 0.103 | 0.49 |
| 50 | G | 0.097 | 0 | 0.082 | 0.00 |
| 51 | A | 0.032 | 21 | 0.066 | 0.21 |
| 52 | H | -0.113 | 2 | 0.127 | 0.02 |
| 53 | L | 0.011 | 70 | 0.096 | 0.70 |
| 54 | S | 0.008 | 70 | 0.057 | 0.70 |
| 55 | L | 0.015 | 57 | 0.069 | 0.57 |
| 56 | R | -0.027 | 25 | 0.051 | 0.25 |
| 57 | G | 0.045 | 15 | 0.079 | 0.15 |
| 58 | L | 0.012 | 53 | 0.065 | 0.53 |
| 59 | P | -0.043 | 13 | 0.084 | 0.13 |
| 60 | V | 0.037 | 40 | 0.106 | 0.40 |
| 61 | C | 0.037 | 21 | 0.068 | 0.21 |
| 62 | A | -0.03 | 19 | 0.062 | 0.19 |
| 63 | F | 0.011 | 76 | 0.081 | 0.76 |
| 64 | S | -0.026 | 36 | 0.082 | 0.36 |
| 65 | S | -0.014 | 58 | 0.084 | 0.58 |
| 66 | A | 0.049 | 18 | 0.115 | 0.18 |
| 67 | G | -0.027 | 38 | 0.074 | 0.38 |
| 68 | P | -0.004 | 81 | 0.073 | 0.81 |
| 69 | C | -0.028 | 39 | 0.097 | 0.39 |
| 70 | A | 0.063 | 9 | 0.159 | 0.09 |
| 71 | L | 0.002 | 94 | 0.071 | 0.94 |
| 72 | R | 0.039 | 20 | 0.081 | 0.20 |
| 73 | F | 0.042 | 29 | 0.093 | 0.29 |
| 74 | T | 0.042 | 26 | 0.111 | 0.26 |
| 75 | S | 0.012 | 74 | 0.103 | 0.74 |
| 76 | A | -0.02 | 42 | 0.093 | 0.42 |
| 77 | R | -0.037 | 8 | 0.057 | 0.08 |
| 78 | R | -0.012 | 57 | 0.085 | 0.57 |
| 79 | M | 0.013 | 66 | 0.085 | 0.66 |
| 80 | E | 0.033 | 39 | 0.107 | 0.39 |
| 81 | T | -0.095 | 1 | 0.095 | 0.01 |
| 82 | T | 0.04 | 2 | 0.042 | 0.02 |
| 83 | V | -0.041 | 8 | 0.066 | 0.08 |
| 84 | N | 0.01 | 71 | 0.092 | 0.71 |
| 85 | A | 0.023 | 49 | 0.125 | 0.49 |
| 86 | H | 0.067 | 3 | 0.077 | 0.03 |
| 87 | R | 0.062 | 6 | 0.082 | 0.06 |
| 88 | N | -0.098 | 2 | 0.109 | 0.02 |
| 89 | L | 0.001 | 98 | 0.078 | 0.98 |
| 90 | P | 0.011 | 28 | 0.027 | 0.28 |
| 91 | K | 0.024 | 47 | 0.063 | 0.47 |
| 92 | V | 0.051 | 3 | 0.06 | 0.03 |
| 93 | L | 0.043 | 9 | 0.062 | 0.09 |
| 94 | H | 0.003 | 86 | 0.121 | 0.86 |
| 95 | K | 0.068 | 4 | 0.075 | 0.04 |
| 96 | R | 0.02 | 51 | 0.078 | 0.51 |
| 97 | T | 0.022 | 50 | 0.103 | 0.50 |
| 98 | L | 0.021 | 48 | 0.073 | 0.48 |
| 99 | G | -0.06 | 7 | 0.098 | 0.07 |
| 100 | L | 0.001 | 93 | 0.047 | 0.93 |
| 101 | S | -0.005 | 93 | 0.106 | 0.93 |
| 102 | A | 0.126 | 0 | 0.086 | 0.00 |
| 103 | M | 0.139 | 0 | 0.075 | 0.00 |
| 104 | S | -0.005 | 79 | 0.059 | 0.79 |
| 105 | T | 0.062 | 2 | 0.073 | 0.02 |
| 106 | T | 0.031 | 12 | 0.044 | 0.12 |
| 107 | D | 0.054 | 0 | 0.051 | 0.00 |
| 108 | L | 0.013 | 60 | 0.072 | 0.60 |
| 109 | E | 0.006 | 79 | 0.066 | 0.79 |
| 110 | A | 0.057 | 0 | 0.054 | 0.00 |
| 111 | Y | -0.071 | 2 | 0.108 | 0.02 |
| 112 | F | 0.026 | 35 | 0.076 | 0.35 |
| 113 | K | 0.006 | 72 | 0.066 | 0.72 |
| 114 | D | 0.04 | 12 | 0.099 | 0.12 |
| 115 | C | -0.009 | 72 | 0.071 | 0.72 |
| 116 | V | 0.002 | 94 | 0.109 | 0.94 |
| 117 | F | 0.019 | 36 | 0.061 | 0.36 |
| 118 | K | 0.222 | 0 | 0.084 | 0.00 |
| 119 | E | -0.051 | 1 | 0.061 | 0.01 |
| 120 | W | 0.012 | 40 | 0.045 | 0.40 |
| 121 | E | 0.016 | 60 | 0.079 | 0.60 |
| 122 | E | 0.028 | 31 | 0.074 | 0.31 |
| 123 | L | -0.084 | 0 | 0.07 | 0.00 |
| 124 | G | 0.09 | 0 | 0.078 | 0.00 |
| 125 | E | 0.29 | 0 | 0.145 | 0.00 |
| 126 | E | 0.03 | 63 | 0.141 | 0.63 |
| 127 | I | 0.073 | 25 | 0.204 | 0.25 |
| 128 | R | 0.17 | 0 | 0.128 | 0.00 |
| 129 | L | 0.21 | 0 | 0.129 | 0.00 |
| 130 | M | 0.101 | 7 | 0.126 | 0.07 |
| 131 | I | 0.077 | 13 | 0.134 | 0.13 |
| 132 | F | 0.038 | 62 | 0.196 | 0.62 |
| 133 | V | -0.027 | 37 | 0.1 | 0.37 |
| 134 | L | -0.158 | 0 | 0.101 | 0.00 |
| 135 | G | 0.001 | 92 | 0.045 | 0.92 |
| 136 | G | 0.009 | 61 | 0.036 | 0.61 |
| 137 | C | 0.018 | 38 | 0.047 | 0.38 |
| 138 | R | 0.056 | 5 | 0.076 | 0.05 |
| 139 | H | 0.001 | 90 | 0.045 | 0.90 |
| 140 | K | 0.019 | 45 | 0.066 | 0.45 |
| 141 | L | -0.027 | 18 | 0.046 | 0.18 |
| 142 | V | 0.02 | 47 | 0.061 | 0.47 |
| 143 | C | 0.082 | 0 | 0.08 | 0.00 |
| 144 | S | -0.148 | 0 | 0.08 | 0.00 |
| 145 | P | -0.02 | 51 | 0.084 | 0.51 |
| 146 | A | 0.116 | 0 | 0.092 | 0.00 |
| 147 | P | 0.039 | 0 | 0.038 | 0.00 |
| 148 | C | 0.039 | 32 | 0.1 | 0.32 |
| 149 | N | 0.022 | 49 | 0.105 | 0.49 |
| 150 | F | 0.016 | 63 | 0.071 | 0.63 |
| 151 | F | 0.044 | 31 | 0.106 | 0.31 |
| 152 | T | 0.093 | 0 | 0.087 | 0.00 |
| 153 | S | -0.072 | 2 | 0.077 | 0.02 |
| 154 | A | 0.02 | 45 | 0.072 | 0.45 |
| 155 | * | 0.314 | 57 | 1.099 | 0.57 |

**User defined p-value: 5/100=0.05. Significant sites with p<=0.05 are highlighted in ● .**
